# A global genomic perspective on the multidrug-resistant *Streptococcus pneumoniae* 15A-CC63 sub-lineage following pneumococcal conjugate vaccine introduction

**DOI:** 10.1099/mgen.0.000998

**Published:** 2023-04-21

**Authors:** Paulina A. Hawkins, Sopio Chochua, Stephanie W. Lo, Sophie Belman, Martin Antonio, Brenda Kwambana-Adams, Anne von Gottberg, Mignon du Plessis, Jen Cornick, Bernard Beall, Robert F. Breiman, Stephen D. Bentley, Lesley McGee, The Global Pneumococcal Sequencing Consortium

**Affiliations:** ^1^​ Respiratory Diseases Branch, Centers for Disease Control and Prevention, Atlanta, GA, USA; ^2^​ Parasites and Microbes, Wellcome Sanger Institute, Hinxton, Cambridge, UK; ^3^​ MRC Unit The Gambia, London School of Hygiene and Tropical Medicine, Banjul, The Gambia; ^4^​ National Institute for Communicable Diseases, National Health Laboratory Service, Johannesburg, South Africa; ^5^​ School of Pathology, University of the Witwatersrand, Johannesburg, South Africa; ^6^​ Malawi–Liverpool–Wellcome Trust Clinical Research Programme, Blantyre, Malawi; ^7^​ Rollins School of Public Health, Emory University, Atlanta, GA, USA

**Keywords:** antimicrobial resistance, pneumococcal conjugate vaccines, pneumococcus, serotype replacement

## Abstract

The introduction of pneumococcal conjugate vaccines (PCV7, PCV10, PCV13) around the world has proved successful in preventing invasive pneumococcal disease. However, immunization against *Streptococcus pneumoniae* has led to serotype replacement by non-vaccine serotypes, including serotype 15A. Clonal complex 63 (CC63) is associated with many serotypes and has been reported in association with 15A after introduction of PCVs. A total of 865 CC63 isolates were included in this study, from the USA (*n*=391) and a global collection (*n*=474) from 1998–2019 and 1995–2018, respectively. We analysed the genomic sequences to identify serotypes and penicillin-binding protein (PBP) genes 1A, 2B and 2X, and other resistance determinants, to predict minimum inhibitory concentrations (MICs) against penicillin, erythromycin, clindamycin, co-trimoxazole and tetracycline. We conducted phylogenetic and spatiotemporal analyses to understand the evolutionary history of the 15A-CC63 sub-lineage. Overall, most (89.5 %, *n*=247) pre-PCV isolates in the CC63 cluster belonged to serotype 14, with 15A representing 6.5 % of isolates. Conversely, serotype 14 isolates represented 28.2 % of post-PCV CC63 isolates (*n*=618), whilst serotype 15A isolates represented 65.4 %. Dating of the CC63 lineage determined the most recent common ancestor emerged in the 1980s, suggesting the 15A-CC63 sub-lineage emerged from its closest serotype 14 ancestor prior to the development of pneumococcal vaccines. This sub-lineage was predominant in the USA, Israel and China. Multidrug resistance (to three or more drug classes) was widespread among isolates in this sub-lineage. We show that the CC63 lineage is globally distributed and most of the isolates are penicillin non-susceptible, and thus should be monitored.

## Data Summary

Raw FastQ data, assemblies and annotations for pneumococcal genomes were previously released to the European Nucleotide Archive (ENA) as part of the Global Pneumococcal Sequencing (GPS) project and the Centers for Disease Control and Prevention (CDC) Active Bacterial Core Surveillance (ABCs) programme, with metadata and accession numbers shown in the Supplementary Material (available with the online version of this article).

Scripts used to run the CDC pneumococcal typing pipeline are available on GitHub: https://github.com/BenJamesMetcalf/Spn_Scripts_Reference. Metadata can be interactively viewed in Microreact: https://microreact.org/project/3aYGgAGBU8Hzey13dHrNXb. More information about the GPS project and ABCs programme can be found at https://www.pneumogen.net/gps/ and https://www.cdc.gov/abcs/overview/background.html, respectively.

**Impact Statement**

Increases in non-pneumococcal conjugate vaccine (non-PCV) serotypes due to expansion of pre-existing clones and emergence of capsule-switch strains have been reported in some countries following the introduction of PCV. In this study, we describe the global distribution of non-vaccine serotype 15A before and after the introduction of PCV. Our findings contribute to the general understanding of pneumococcal molecular epidemiology in the PCV era, as well as the international effort to characterize replacement serotypes.

## Introduction

The introduction of pneumococcal conjugate vaccines (PCV7, PCV10, PCV13) in countries around the world has proved successful in the prevention of invasive pneumococcal disease. However, immunization against *Streptococcus pneumoniae* has led to serotype replacement by non-vaccine serotypes (NVTs), including serotype 15A, and a concomitant increase in antimicrobial resistance associated with some of these serotypes [1–6]. In some cases, serotype replacement has occurred through capsular switching, but most often it is due to the expansion of previously recognized clones of NVTs. The expansion of the multidrug-resistant 15A/sequence type (ST)63 (Sweden^15A^-ST63, PMEN25) and related clones (clonal complex 63; CC63) after the introduction of PCVs has been reported in several countries, both among carriage and invasive pneumococcal isolates: Canada [7], Germany [8], Japan [9], Norway [10], the USA [11–13] and Taiwan [14].

The Global Pneumococcal Sequencing (GPS) project is a whole-genome sequencing survey of the global pneumococcal population in the context of PCV use. The Centers for Disease Control and Prevention (CDC) Active Bacterial Core Surveillance (ABCs) programme is an active laboratory and population-based surveillance system in the USA for invasive bacterial pathogens of public-health importance, including *S. pneumoniae *. In this study, we examined the global distribution of the 15A-CC63 sub-lineage in the context of these two large isolate collections.

## Methods

### Isolates

A total of 865 isolates were included in this study from the USA (*n*=391) and a global collection (*n*=474) spanning years 1998–2019 [15–19] and 1995–2018 [20], respectively. Of these, 695 isolates were sourced from disease samples, the rest (170) were carriage isolates. All isolates belonged to CC63, comprising ST63 (*n*=515), single locus variants (*n*=256) of ST63 and double locus variants (*n*=94) of ST63. Most isolates (98.3 %, *n*=850) belonged to the previously described global pneumococcal sequence cluster GPSC9 [20]. Country of origin, vaccine period, age and source information for all isolates are shown in Table 1.

**Table 1.** Country of origin, vaccine period, age and source information for all isolates

Country	Year of vaccine introduction (valence)	*n*	Serotype (by PCV introduction status)								Source of specimen		Age of cases (years)	
			Pre-PCV				Post-PCV							
			**Total**	14 [*n* (%)]	15A [*n* (%)]	Other [*n* (%)]	Total	14 [*n* (%)]	15A [*n* (%)]	Other [*n* (%)]	Disease	Carriage	5 and under	Older than 5
Argentina	2012 (PCV13)	16	8	7 (88)	0 (0)	1 (12)	8	8 (100)	0 (0)	0 (0)	16	0	16	0
Bangladesh	2015 (PCV10)	31	29	29 (100)	0 (0)	0 (0)	2	2 (100)	0 (0)	0 (0)	30	1	22	9
Cambodia	2015 (PCV13)	60	16	14 (87)	2 (13)	0 (0)	44	31 (70)	13 (30)	0 (0)	23	37	60	0
China (Hong Kong)	2009 (PCV7) 2011 (PCV13)	18	7	0 (0)	7 (100)	0 (0)	11	0 (0)	11 (100)	0 (0)	3	15	17	1
India*	2017 (PCV10)	58	45	45 (100)	0 (0)	0 (0)	13	13 (100)	0 (0)	0 (0)	46	12	30	15
Israel	2009 (PCV7) 2011 (PCV13)	16	2	0 (0)	1 (50)	1 (50)	14	0 (0)	13 (93)	1 (7)	16	0	11	5
Kenya†	2011 (PCV10)	10	4	4 (100)	0 (0)	0 (0)	6	6 (100)	0(0)	0(0)	4	6	–	–
Malawi	2011 (PCV13)	33	26	26 (100)	0 (0)	0(0)	7	7 (100)	0 (0)	0 (0)	14	19	24	9
Mozambique†	2013 (PCV10)	24	24	24 (100)	0 (0)	0 (0)	0	–	–	–	24	0	–	–
Nepal	2015 (PCV10)	22	22	22 (100)	0 (0)	0 (0)	0	–	–	–	12	10	22	0
Nigeria	2014 (PCV10)	11	0	–	–	–	11	11 (100)	0 (0)	0 (0)	11	0	11	0
South Africa	2009 (PCV7) 2011 (PCV13)	69	26	26 (100)	0 (0)	0 (0)	43	35 (81)	8 (19)	0 (0)	48	21	58	11
Thailand	No PCV‡	25	25	11 (44)	6 (24)	8 (32)	0	–	–	–	25	0	17	8
The Gambia*	2009 (PCV7) 2011 (PCV13)	81	9	9 (100)	0 (0)	0 (0)	72	56 (78)	0 (0)	16 (22)	32	49	64	5
USA (ABCs)	2000 (PCV7) 2010 (PCV13)	391	4	4 (100)	0 (0)	0 (0)	387	5 (1)	359 (93)	23 (6)	391	0	38	353

*Some isolates are missing age information.

†No age data.

‡PCV10 and PCV13 are available in Thailand, but not included in the National Immunization Programme.

### Genomic analyses

Genomic DNA from all isolates was sequenced on the Illumina HiSeq or MiSeq platforms to produce paired-end reads [15–18]. European Nucleotide Archive (ENA) accession numbers can be found in Table S1. Genomic sequences were analysed using the CDC *Streptococcus* laboratory pneumococcal typing pipeline; the methods and programs used have been previously described [15–19], with periodic updates at: https://github.com/BenJamesMetcalf/Spn_Scripts_Reference. We identified penicillin-binding protein (PBP) genes 1A, 2B and 2X, and other resistance determinants, to predict minimum inhibitory concentrations (MICs) against β-lactams, erythromycin, clindamycin, co-trimoxazole and tetracycline [21, 22]. We used non-meningitis penicillin breakpoints (≤0.06, 0.12–1 and ≥2 µg l^−1^), and defined susceptibility and non-susceptibility as MICs of ≤0.06 and ≥0.12 µg l^−1^, respectively. Erythromycin and clindamycin susceptibility, intermediate resistance and resistance were defined as MICs of ≤0.25, 0.5 and ≥1 µg l^−1^, respectively. Co-trimoxazole susceptibility, intermediate resistance and resistance were defined as MICs of ≤0.5, 1–2 and ≥4 µg l^−1^, respectively. Tetracycline susceptibility, intermediate resistance and resistance were defined as MICs of ≤1, 2 and >4 µg l^−1^, respectively.

PHYLOViZ [23] was used to create a minimum spanning tree of allelic profiles, using a 10-loci scheme that combined MLST (multilocus sequence type) (https://pubmlst.org/) and PBP type, as previously described [16, 17]. SNP analysis was performed using kSNP [24]. RAxML (Randomized Axelerated Maximum Likelihood) [25] was used to build maximum-likelihood phylogenetic trees. Microreact was used to visualize the resulting trees and associated metadata [26].

Recombination was detected and removed using Gubbins (Genealogies Unbiased By recomBinations In Nucleotide Sequences) [27]. The resulting tree and recombination predictions, along with corresponding metadata, were visualized using Phandango [28]. Branch length was then converted to time since most recent common ancestor (MRCA) using BactDating, with a mixed gamma, relaxed clock model [29]. The effective sample size was over 200 for the inferred parameters α, μ and σ.

The chi-square test was used for all statistical comparisons.

## Results and Discussion

### Serotype and genotype

**Fig. 1.**
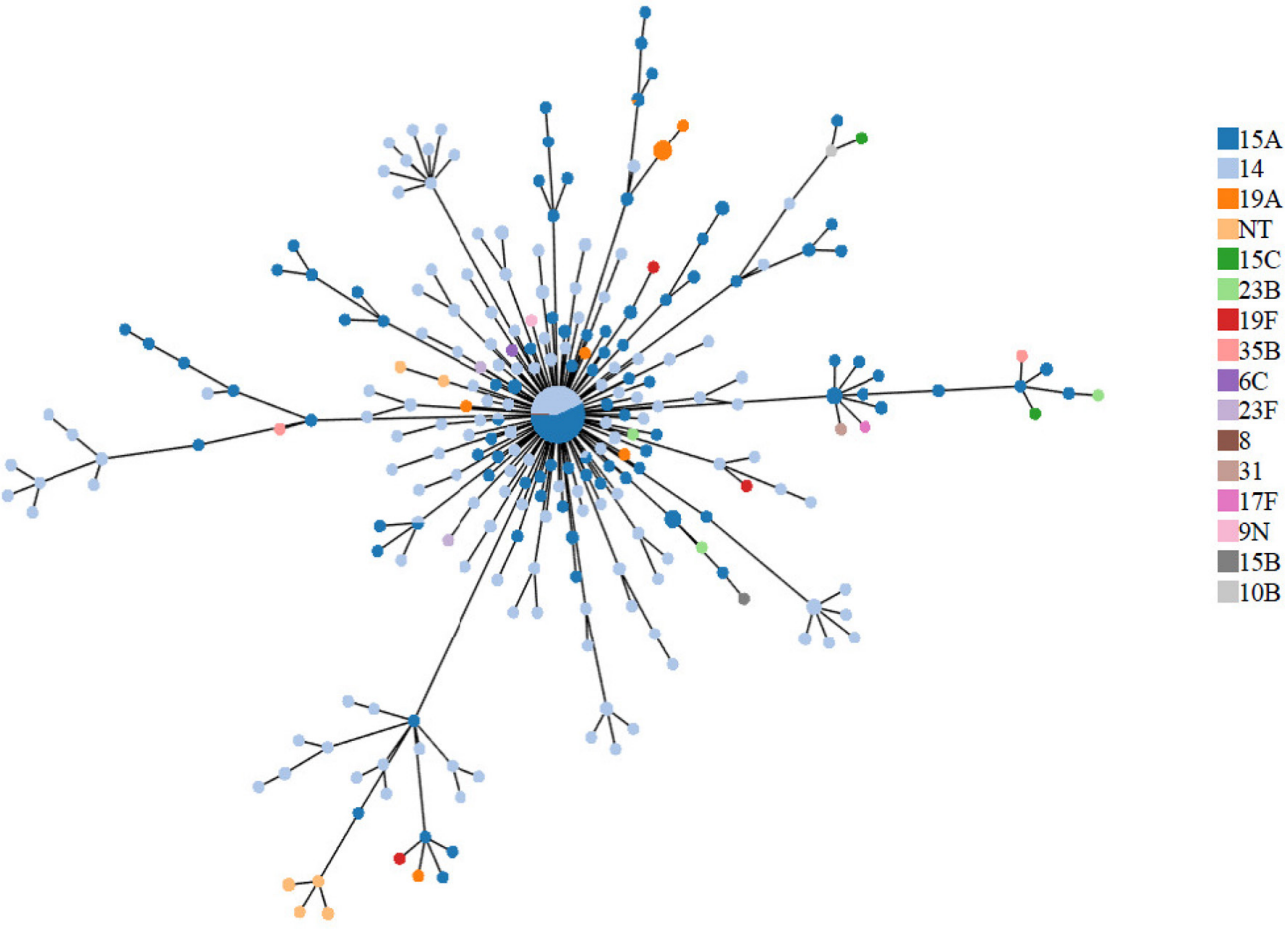
Minimum-spanning-tree of all isolates (*n*=865) using a 10-loci scheme (7 MLST alleles plus 3 PBP alleles). The size of each circle is proportional to the number of isolates with that allele combination, and the colour represents the proportion of each serotype. For each tip, the distance from the centre represents the number of alleles that are different from ST63/PBP-type 24-27-28. NT, Non-typeable.

A total of 104 variants of ST63 were identified: 70 single locus variants and 34 double locus variants, representing 15 serotypes: 6C, 8, 9N, 10B, 14, 15A, 15B, 15C, 17F, 19A, 19F, 23B, 23F, 31, 35B. The majority (59.5 %, *n*=515 out of 865) of the isolates belonged to ST63, and most were either serotype 15A (48.6 %, *n*=420) or 14 (45.7 %, *n*=395). Except for ST63, all STs were associated with a single serotype (Fig. 1).

Serotype 14 was the most common among carriage isolates (*n*=124, 72.9 %), while 56.3 % (*n*=391) of disease isolates were serotype 15A. However, this is most likely due to geographical origin, since most disease 15A isolates were from the USA (*n*=361, 92.3 %), where serotype 15A predominates among ST63 isolates.

**Fig. 2.**
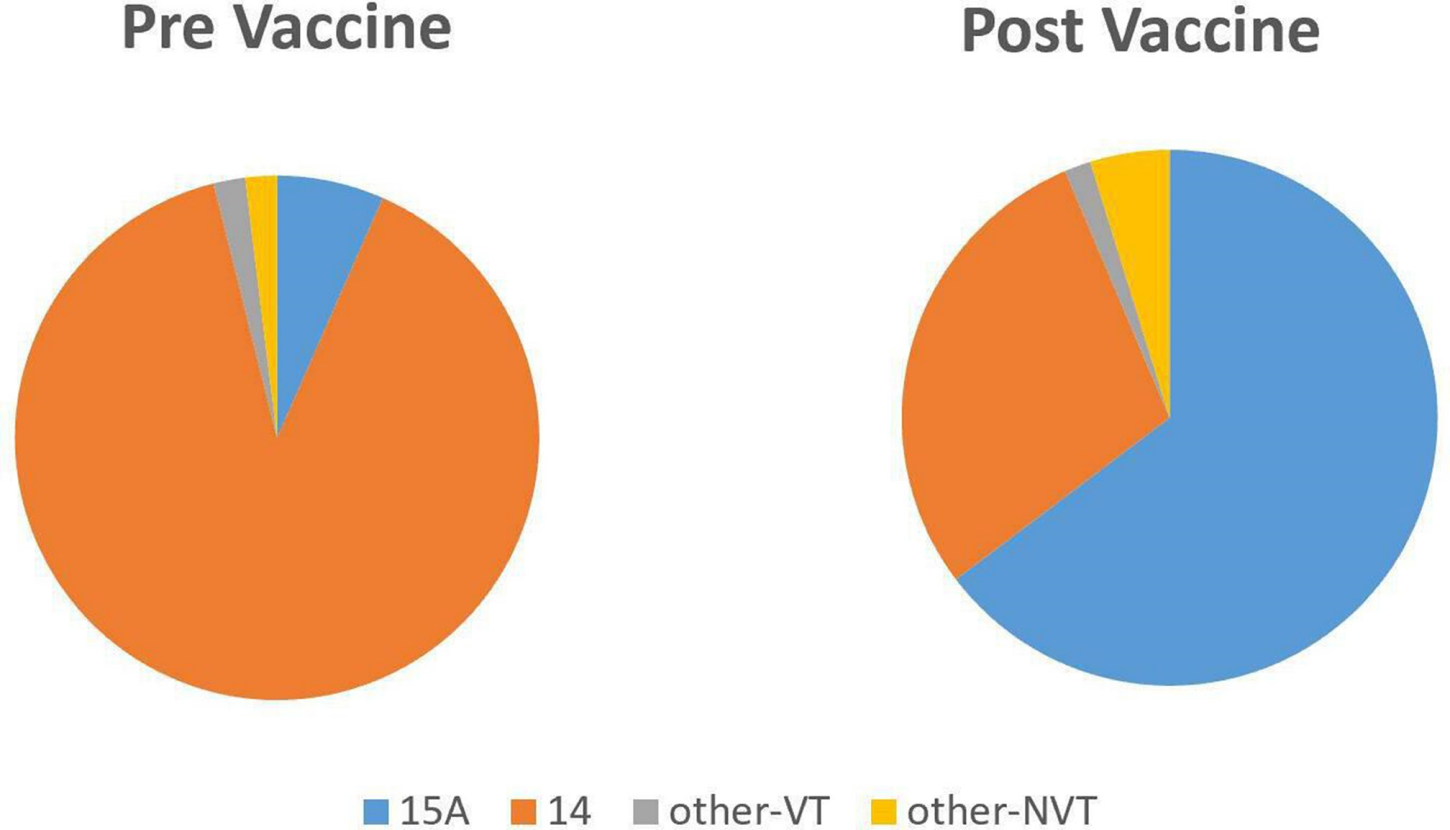
Serotype distribution before (*n*=247) and after (*n*=618) vaccine introduction (any PCV), grouped into vaccine types (14 plus other-VT) and non-vaccine types (15A plus other-NVT). Most (89.5%) of the pre-PCV isolates belonged to serotype 14, with 15A representing only 6.5 % of isolates. Conversely, serotype 14 isolates represented only 28.2 % of post-PCV CC63 isolates, while serotype 15A isolates represented 65.4 % (*P*<0.000001).

A comparison of overall serotype distributions among the study isolates from before and after the introduction of one of the PCVs (PCV7, PCV10 or PCV13), revealed a decline in the proportion of serotype 14 and concurrent increase in the proportion of serotype 15A (Fig. 2). Most (89.5 %, *n*=221) of the pre-PCV isolates (*n*=247) in the CC63 cluster belonged to serotype 14, with 15A representing only 6.5 % (*n*=16) of isolates. Conversely, serotype 14 isolates represented only 28.2 % (*n*=174) of post-PCV CC63 isolates (*n*=618), whilst serotype 15A isolates represented 65.4 % (*n*=404).

Overall, serotype 14-CC63 isolates were predominant in Argentina, Sub-Saharan Africa (Kenya, Malawi, Mozambique, Nigeria, South Africa and The Gambia), the Indian subcontinent (India, Bangladesh and Nepal), and South-East Asia (Cambodia and Thailand), whilst the 15A-CC63 sub-lineage was predominant in the USA, Israel and China (Hong Kong).

All isolates from the Indian subcontinent (*n*=111) belonged to the 14-CC63 sub-lineage. Since PCVs were introduced in Nepal and Bangladesh in 2015, and in India in 2017, the majority (86.5 %) of our samples from this region predated vaccines. Serotype 14-CC63 was also the predominant sub-lineage in Sub-Saharan Africa, where serotype 14 was expressed by all pre-PCV CC63 isolates (*n*=89).

In The Gambia, PCV7 was introduced in 2009 and replaced by PCV13 in 2011. The proportion of serotype 14 (all genotypes) among Gambian isolates in the GPS collection remained stable during the pre-, post-PCV7 and post-PCV-13 periods (2–4 %). However, the proportion of ST63 isolates among serotype 14 isolates increased from about 50 % in the pre-PCV and PCV7 periods, to 94 % (*n*=35) in the post-PCV13 period.

Like The Gambia, South Africa implemented PCV7 in its routine immunization programme in 2009 and transitioned to PCV13 in 2011, but in contrast, the introduction of PCVs in South Africa had a marked effect on serotype 14 incidence, which decreased significantly from 2008 to 2011 [30]. We first observed serotype 15A among South African study isolates collected in 2011 (12.5 %, *n*=8), whilst all isolates from 2013 (*n*=5), when vaccine coverage in South Africa had reached an estimated 87 % [31], belonged to serotype 15A.

In Cambodia, where PCV13 was introduced in 2015, 89.3 % (*n*=28) of CC63 isolates belonged to serotype 14 in 2015–2016, but this proportion decreased to 37.5 % (*n*=16) in 2017, with the rest belonging to serotype 15A. In contrast, in Thailand, where PCVs are available but not part of the national immunization programme [32], serotypes 14 and 15A were evenly represented among our sample of CC63 isolates (44 and 40 %, respectively; *n*=25).

The 15A-CC63 sub-lineage was most predominant in the USA, Israel and China. In Israel, 92.8 % (*n*=14) of post-PCV CC63 isolates belonged to serotype 15A, whilst in China (Hong Kong), all pre- and post-PCV CC63 isolates (*n*=18) were 15A.

Among CC63 isolates from the USA, 64.3 % (*n*=14) post-PCV7 (2001–2009) and 94.9 % (*n*=373) post-PCV13 (2010–2019) belonged to serotype 15A. Our sampling only included four isolates from the pre-PCV period (all serotype 14), mainly because CC63 isolates and 15A isolates of all genotypes were rare in the USA pre-PCV7. Only 8 serotype 15A isolates were collected in 1999, out of 4547 total ABCs samples (surveillance population of 18 550 681) and only 2 of them were CC63. Invasive pneumococcal disease plummeted, but serotype 15A incidence increased modestly, after PCV7 was introduced, then levelled off before PCV13 was introduced, and has remained stable since then [11] (unpublished ABCs data). In 2009, 109 serotype 15A isolates (99 of them CC63, 90.8 %) were found among 4176 ABCs isolates (surveillance population of 29 206 528).

**Fig. 3.**
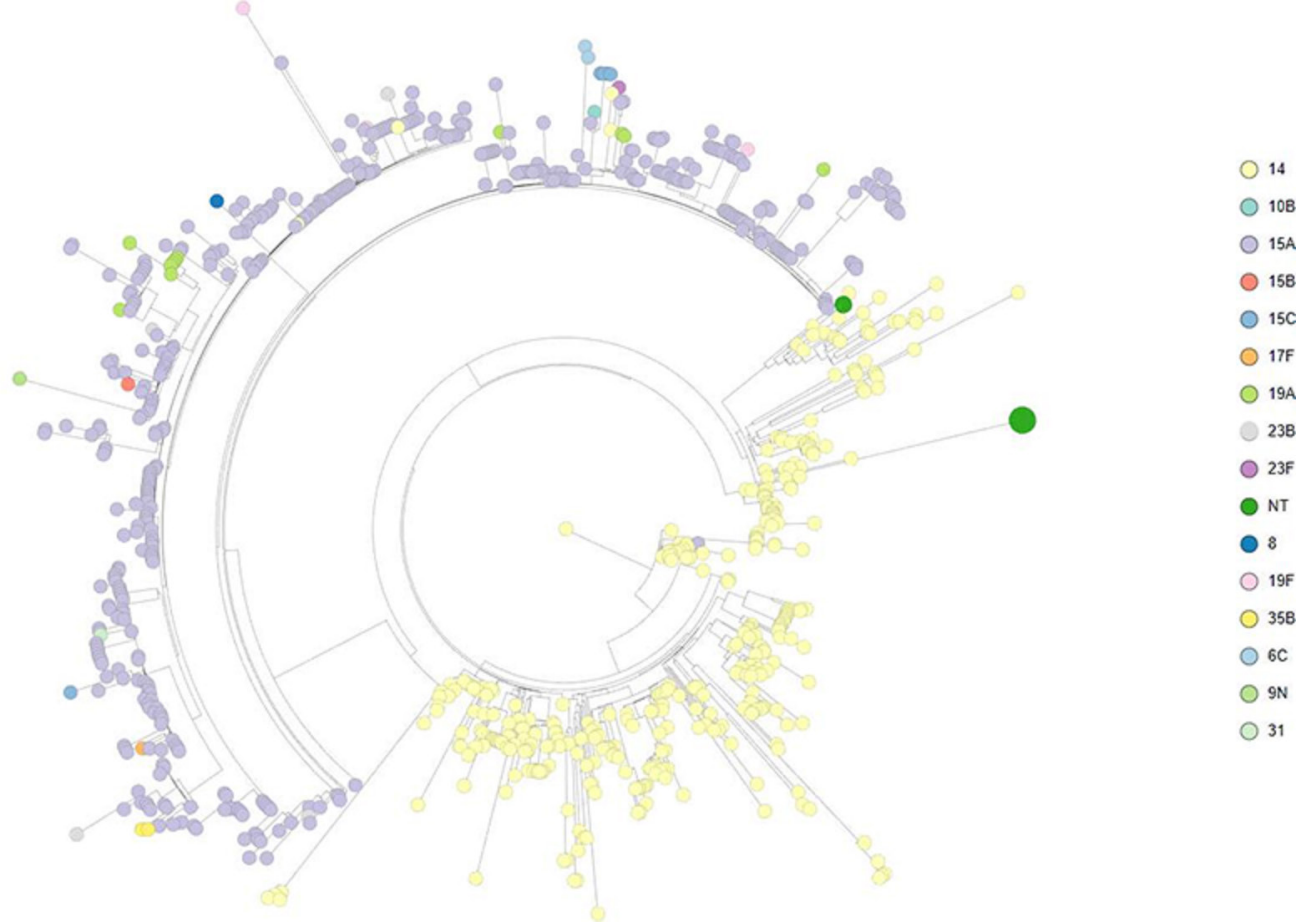
Phylogenetic tree of all CC63 isolates (*n*=865) prior to recombination analysis. Each colour represents a different serotype. Two branches of non-typeable (NT) isolates (in green) were collapsed; the size of the tips is proportional to the number of isolates in each branch. Two very homogenous clades of serotype 14 isolates can be observed, along with a single clade of PMEN25-related isolates.

Fig. 3 shows a phylogenetic tree of all the study isolates. The serotype 14 clades are very homogeneous, except for two branches of non-typeable carriage isolates from The Gambia. The first branch is composed of two isolates (ST12149 and ST63) with total absence of *cps* genes and identical *pbp*2x-*dex*B*–ali*A-*pbp*1a sequences (Supplementary Data S1); most closely related to an ST3310 isolate. The second branch is composed of 14 isolates (ST4040 and ST10967) without *cps* genes (Supplementary Data S2), and with a *dex*B—*ali*A region that most likely emerged from recombination with the previously described NT lineage ST344/GPSC81 (98.8 % identity) [33].

The 15A-CC63 sub-lineage (*n*=454) includes 13 additional serotypes (6C, 8, 9 N, 10B, 15B/C, 17A, 19A, 19F, 23B, 23F, 31, 35B). Most of the non-15A isolates in this clade (35 of 38) were from the USA or Thailand.

Of interest, the serotype 8-ST63 combination was previously determined to be a recombination of a highly invasive 8-ST53 clone and the multidrug-resistant Sweden^15A^-ST63 clone, and widespread in Spain as of 2014 [34]; a single representative of this lineage, from the ABCs collection, was included in our study [16]. The serotype 19A-ST63 combination, which has previously been reported in Portugal [35], Australia [36] and Italy [37], was identified in 10 isolates from Thailand (*n*=2), Israel (*n*=1) and the USA (*n*=7); 6/7 USA isolates were isolated post-PCV13. Additionally, four isolates from Thailand belonged to the 15C-ST8346 lineage, which has been previously reported in Japan [38].

### Recombination and phylogenetic dating

**Fig. 4.**
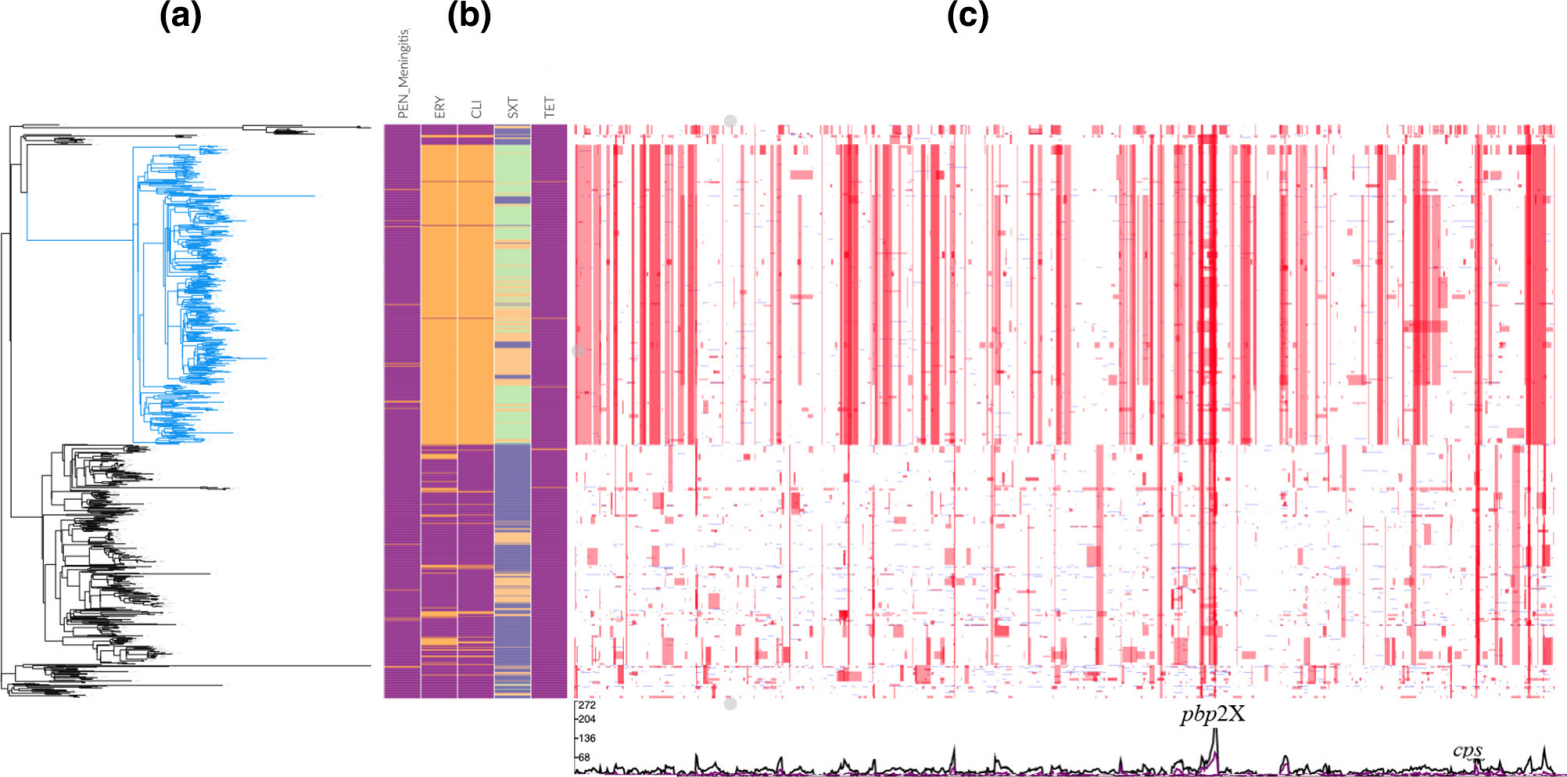
**(a**) Maximum-likelihood tree inferred from non-recombinant regions, with the PMEN25 clade in blue and serotype 14 clades in black. (**b**) Associated-resistance data: penicillin (PEN), erythromycin (ERY) and tetracycline (TET) resistance shown in magenta (susceptible) and orange (non-susceptible); co-trimoxazole (SXT) resistance shown in purple (resistant), orange (intermediate) and green (susceptible). (**c**) Putative recombination events detected in each terminal taxon. Red blocks are recombination events predicted to have occurred on an internal branch and, therefore, are shared by multiple isolates through common descent. Blue blocks are recombination events predicted to occur on terminal branches and, hence, are present in only one strain. Overlapping blocks increase the intensity of the colours. At the bottom, in the graph, the line represents the frequency of recombination events along the length of the genome, with the *pbp2X* and *cps* loci labelled.

Fig. 4(a, b, c) shows the results of the recombination analysis, including a maximum-likelihood phylogeny reconstructed from the non-recombinant regions of the alignment (Fig. 4a). A total of 129 395 SNPs were identified. Several recombination hotspots were evident in the genome, but the most notable was in *pbp*2X, which reflected the diversity of PBP types that were identified (Fig. 4c).

**Fig. 5.**
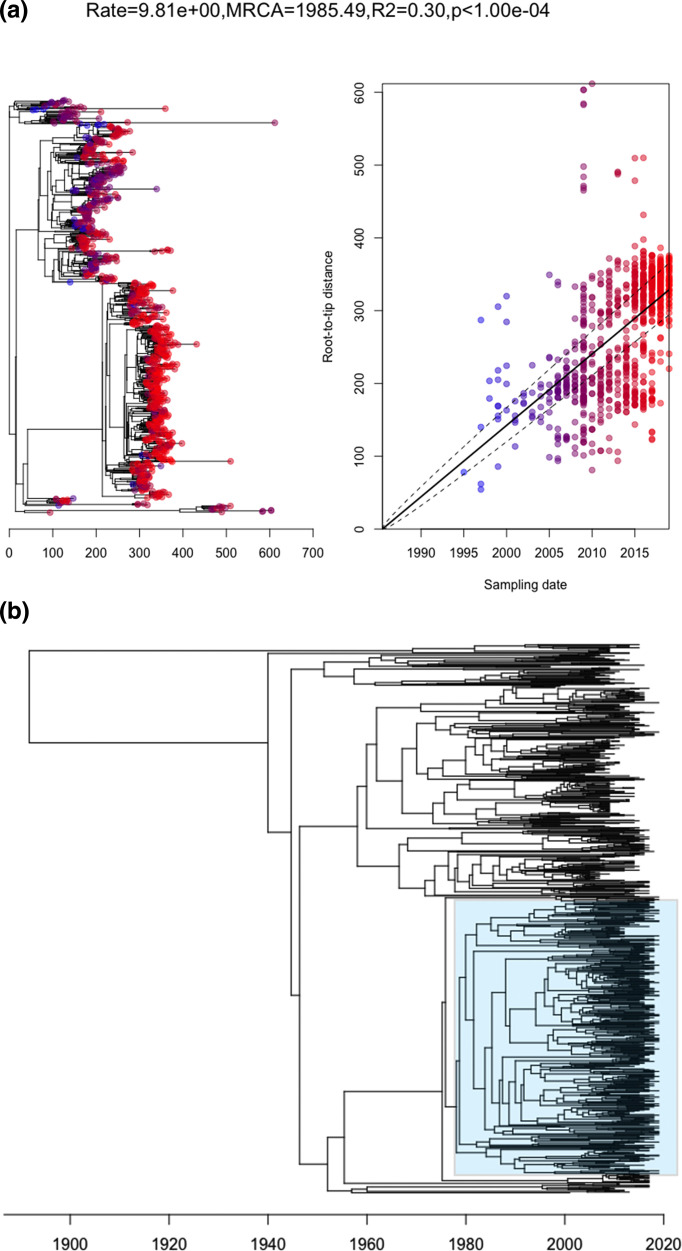
(a) Root-to-tip regression analysis to detect evidence of a temporal signal among all CC63 isolates. The left panel shows the recombination-free tree from which the root-to-tip distance was calculated, with tips shaded in a colour gradient from red (more recent) to blue (older) isolates. The right panel shows the regression of root-to-tip distance on the year of collection; with dashed lines representing CIs. This regression suggested a significant temporal signal in the data. (**b**) Dated phylogeny reconstruction using BactDating, with the PMEN25 clade shaded in blue. The date of the most recent common ancestor was estimated to be around 1981 (95% CI 1972–1989).

Root-to-tip analysis showed a significant temporal signal, with *R* ^2^=0.30 and *P*<0.001, allowing for coalescent analysis (Fig. 5a). A reconstruction of the dated phylogeny (Fig. 5b) suggested the 15A-CC63 sub-lineage emerged from its closest serotype 14 ancestor around 1981 [95 % confidence interval (CI) 1972–1989], prior to the introduction of pneumococcal vaccines. The 95 % CIs for ancestral dates are represented by blue bars on the dated phylogeny in Fig. S1. The inferred parameters were: mean substitution rate μ=2.89 substitutions per site (95 % CI 2.66–3.14), standard deviation of the per-branch substitution rates σ=6.20 substitutions per site (95 % CI 5.58–6.84), and coalescence time unit α=40.16 generations (95 % CI 35.56–45.39).

### Antimicrobial resistance in the 15A-CC63 sub-lineage

Multidrug resistance, defined as resistance to three or more drug classes, was widespread among isolates in this sub-lineage (*N*=454) (Fig. 5b). Most notably, 98.0 % (*n*=445) of isolates were predicted to be penicillin non-susceptible (PNS; predicted MIC range 0.12–2 µg ml^−1^); of these, 40 (9.0 %) were also predicted as non-susceptible to meropenem (MIC=0.5 µg/l). A high prevalence of PNS has been previously reported in the 15A-CC63 sub-lineage after vaccine introduction [8, 11, 13–18, 39–42].

A total of 82 PBP types was identified among isolates in this sub-lineage (166 in total among all isolates in the study), mostly driven by diversity in *pbp2X*, with 184 (40.5 %) of the isolates represented by the 24-27-28 combination (Table S2). Only nine of these PBP types were associated with multiple STs and serotypes, the rest were associated with a single ST and serotype. Except for three combinations (24-27-28, 24-27-8 and 34-89-147), each PBP type was identified in isolates from a single country.

Full resistance (MIC=2 µg μl^−1^) was predicted for nine PBP types (present in 39 isolates) identified in this sub-lineage. These included the 4-7-7 and 4-74-7 combinations, which were likely acquired by recombination with a 35B-ST558 strain, as described by Metcalf *et al*. [16]. Of these, seven PBP types were associated with serotype 15A, one with 19A and one with 19F. By comparison, 13 of the PBP types in the 14-CC63 sub-lineage were predicted to confer full resistance to penicillin (present in 30 isolates); 10 of these 13 combinations remained in the post-PCV era.

In addition, 99.1 % of the isolates in the 15A-CC63 sub-lineage carried the *ermB* and *tetM* genes, and were thus predicted to be resistant to erythromycin, clindamycin and tetracycline. The *erm*B gene was carried in a *Tn*917 cassette [43], inserted into a *Tn*916-like element carrying *tet*M [44]; this element was highly conserved within this clade. A similar element has been previously identified in *S. pneumoniae* [45].

Nonsusceptibility to cotrimoxazole, due to changes within *folA* and/or *folP*, was less prevalent, with 60.2 % of the 15A-CC63 sub-lineage isolates predicted as susceptible, 33.0 % as intermediate and only 6.8 % as resistant. Less common resistance genotypes included ten isolates carrying the *aph(3’)-III* gene, which confers resistance to aminoglycosides [46], and one isolate with changes in the *rpoB* gene, which confers resistance to rifampin [20]. An additional eight isolates had changes in *parC*, which confers reduced susceptibility to fluoroquinolones, most often reflected by clinically relevant MICs for ciprofloxacin.

Limitations to this study include: varied sampling strategies and timings of vaccine introduction among different countries, lack of post-vaccine sampling in some of the countries included in the GPS collection, and low overall proportion of CC63 isolates among GPS isolates (2.0 %) and ABCs isolates (2.6 %) for the years tested.

We conclude that because the CC63lineage is globally distributed and most of these isolates are PNS, it merits continued close monitoring. The 15A-CC63 sub-lineage in particular, since it is composed almost exclusively (95.8 %) of non-vaccine type multidrug-resistant isolates, has a high invasive potential [19], and is not included in either of the most recently licensed PCVs (PCV15 and PCV20).

## Supplementary Data

Supplementary material 1
