# Supplementary material for: A global genomic perspective on the multidrug-resistant Streptococcus pneumoniae 15A-CC63 sub-lineage following pneumococcal conjugate vaccine introduction
Source: Microb Genom. 2023 Apr 21;9(4):mgen000998. doi: 10.1099/mgen.0.000998 (PMC10210946; doi:10.1099/mgen.0.000998)
Supplement: Supplementary material 1 [file mgen-9-998-s001.pdf]

Table S1. ENA Accession numbers

Isolate Name	Accession #
20152153	SAMN05220890
20152159	SAMN05220895
20152241	SAMN05220918
20152253	SAMN05220930
20152653	SAMN05220992
20152687	SAMN05221017
20152831	SAMN05221079
20152909	SAMN05221112
20152957	SAMN05221134
20153860	SAMN05221421
20153913	SAMN05221459
20153927	SAMN05221473
20153959	SAMN05221505
20153961	SAMN05221507
20154443	SAMN05221948
20154492	SAMN05221995
20154523	SAMN05222025
20154582	SAMN05222041
20154586	SAMN05222045
20154736	SAMN05222098
20154754	SAMN05222114
20154767	SAMN09224510
20154875	SAMN05222151
20154988	SAMN05222227
20155010	SAMN05222249
20155246	SAMN05222278
20155247	SAMN05222279
20155284	SAMN05222299
20155297	SAMN05222312
20155306	SAMN05222321
20155311	SAMN05222326
20155350	SAMN05222364
20155476	SAMN05222421
20155489	SAMN05222432
20155523	SAMN05222466
20155569	SAMN05222511
20155964	SAMN09224376
20155969	SAMN09231458
20156074	SAMN05222552
20156093	SAMN05222571
20156146	SAMN05222580
20156290	SAMN05222592
20156655	SAMN05222682
20156667	SAMN05222694

Table S2. PBP types identified among all isolates

PBP-type	Serotypes	Penicillin susceptible	n
24--27--28	8, 14, 15A, 19A, 23B	No	326
24--27--13	15A	No	39
24--27--11	15A	No	32
67--27--35	15A, 19A	No	15
17--233--43	15A	No	14
24--27--179	15A	No	13
34--89--147	15A	No	13
24--27--35	14, 15A	No	13
24--27--143	15A	No	10
24--27--8	14, 15A	No	8
23--27--28	15A	No	7
13--31--114	15A	No	6
79--27--35	19A	No	4
250--27--407	15C	No	4
24--27--114	15A	No	4
24--27--73	15A	No	4
24--31--13	15A	No	4
13--175--43	15A	No	3
148--7--138	15A	No	3
24--31--8	15A	No	3
24--27--203	14, 15A	No	3
24--27--0	10B, 14, 15A	Yes	3
2--27--28	6A	No	2
2--27--6	19A	Yes	2
24--62--11	15A, 15B	No	2
13--7--73	15A	No	2
148--7--390	15A	No	2
189--27--28	15A	No	2
24--27--131	15A	No	2
24--27--138	15A	No	2
24--27--181	15A	No	2
24--31--114	15A	No	2
24--62--73	15A	No	2
24--7--73	15A	No	2
34--27--56	15A	No	2
4--7--7	15A	No	2
24--27--109	14, 15A	No	2
24--27--205	14, 15A	No	2
1--27--2	9N	Yes	1
12--27--13	31	No	1
4--27--114	35B	No	1
4--31--114	35B	No	1
15--27--369	23F	No	1
0--27--11	23B	No	1

20156685	SAMN05222712	167--27--1	23B	No	1
20156692	SAMN05222718	7--31--8	23B	No	1
20156760	SAMN05222759	147--229--8	19F	No	1
20160272	SAMN05222827	24--27--192	19F	No	1
20160442	SAMN05222867	8--230--143	19F	No	1
20160523	SAMN05222875	191--229--8	19A	No	1
20160539	SAMN05222890	24--27--16	19A	No	1
20160557	SAMN05222908	0--0--28	19A	Yes	1
20160583	SAMN05222934	0--27--13	17F	No	1
20160587	SAMN05222938	24--31--9	15C	No	1
20160732	SAMN05222951	146--27--7	15A	No	1
20160898	SAMN05222981	148--7--2	15A	No	1
20161055	SAMN05223025	192--229--8	15A	No	1
20161149	SAMN05223047	207--27--13	15A	No	1
20161151	SAMN05223049	23--143--28	15A	No	1
20161192	SAMN05223090	24--16--138	15A	No	1
20161251	SAMN09230968	24--229--8	15A	No	1
20161294	SAMN09231326	24--233--43	15A	No	1
20161366	SAMN05223111	24--27--139	15A	No	1
20161387	SAMN05223130	24--27--147	15A	No	1
20161396	SAMN05223139	24--27--165	15A	No	1
20161618	SAMN05223193	24--27--223	15A	No	1
20161634	SAMN05223209	24--27--232	15A	No	1
20161647	SAMN05223222	24--27--238	15A	No	1
20161656	SAMN05223231	24--27--369	15A	No	1
20161711	SAMN09223882	24--27--374	15A	No	1
20161801	SAMN09223885	24--27--382	15A	No	1
20161807	SAMN09223934	24--27--391	15A	No	1
20161815	SAMN09231107	24--27--43	15A	No	1
20161820	SAMN09224167	24--27--77	15A	No	1
20161996	SAMN09231241	24--27--971	15A	No	1
20162018	SAMN09231075	24--27--973	15A	No	1
20162042	SAMN09218902	24--7--114	15A	No	1
20162389	SAMN09223939	24--73--114	15A	No	1
20162794	SAMN09218797	4--7--114	15A	No	1
20162883	SAMN09224619	4--74--7	15A	No	1
20162921	SAMN09224306	67--73--35	15A	No	1
20162956	SAMN09223940	84--229--368	15A	No	1
20163017	SAMN09231291	55--27--418	NT	No	9
20163114	SAMN09224140	349--479--418	NT	No	4
20163118	SAMN09224841	86--27--418	NT	No	1
20163125	SAMN09219394	348--27--787	NT	No	2
20163127	SAMN09224141	24--53--77	14	No	33
20163182	SAMN09223896	24--27--242	14	No	19
20163217	SAMN09219393	24--27--435	14	No	13
20163451	SAMN09223899	24--27--417	14	No	9
20163470	SAMN09224278	24--27--445	14	No	9

20163478	SAMN09231381	13--309--8	14	No	8
20163506	SAMN09231466	24--384--651	14	No	8
20163650	SAMN09223901	24--73--554	14	No	8
20163659	SAMN09231345	24--27--171	14	No	7
20163662	SAMN09218978	24--27--19	14	No	7
20163713	SAMN09219403	24--27--429	14	No	6
20163729	SAMN09218647	24--27--411	14	No	5
20164091	SAMN09224174	24--27--419	14	No	5
20164160	SAMN09219128	24--27--466	14	No	5
20164273	SAMN09224416	17--228--205	14	No	4
20164310	SAMN09219047	24--27--420	14	No	4
20164312	SAMN09218555	24--27--426	14	No	4
20164351	SAMN09224759	24--262--19	14	No	3
20164355	SAMN09218474	24--27--421	14	No	3
20164392	SAMN09219116	24--27--423	14	No	3
20164403	SAMN09224060	24--27--91	14	No	3
20164414	SAMN09219294	329--451--8	14	No	3
20164431	SAMN09218614	35--33--36	14	No	3
20164434	SAMN09219308	13--310--47	14	No	2
20164448	SAMN09219275	13--385--524	14	No	2
20164514	SAMN09224685	13--406--8	14	No	2
20164571	SAMN09218920	147--73--8	14	No	2
20164811	SAMN09218943	15--263--205	14	No	2
20164836	SAMN09224658	24--27--410	14	No	2
20165167	SAMN09218760	24--27--427	14	No	2
20165169	SAMN09218855	24--27--430	14	No	2
20165195	SAMN09218802	24--27--438	14	No	2
20165461	SAMN09218906	24--27--651	14	No	2
20165462	SAMN09219217	24--27--812	14	No	2
20165520	SAMN09224559	326--444--727	14	No	2
20165570	SAMN09218596	34--73--554	14	No	2
20165643	SAMN09218762	13--387--20	14	No	1
20165656	SAMN09218638	13--73--554	14	No	1
20165895	SAMN09224831	13--73--73	14	No	1
20165897	SAMN09218600	147--27--8	14	No	1
20165981	SAMN09218909	17--389--8	14	No	1
20165987	SAMN09231509	17--491--8	14	No	1
20166394	SAMN09219127	24--237--385	14	No	1
20166571	SAMN09218640	24--265--434	14	No	1
20166573	SAMN09231264	24--27--135	14	No	1
20166792	SAMN09230953	24--27--177	14	No	1
20166835	SAMN09218668	24--27--20	14	No	1
20166847	SAMN09231445	24--27--22	14	No	1
20170077	SAMN09224530	24--27--36	14	No	1
20170082	SAMN09231396	24--27--388	14	No	1
20170474	SAMN09223911	24--27--409	14	No	1
20170475	SAMN09224024	24--27--413	14	No	1

20170481	SAMN09231222
20170722	SAMN09218525
20170725	SAMN09224288
20170728	SAMN09218653
20170741	SAMN09224773
20170787	SAMN09219398
20170838	SAMN09218459
20170840	SAMN09224256
20170938	SAMN09224395
20170948	SAMN09218884
20171132	SAMN09224734
20171232	SAMN09224605
20171445	SAMN09218748
20171472	SAMN09219083
20171615	SAMN09224835
20171630	SAMN09224811
20171648	SAMN09223876
20171662	SAMN09218463
20171669	SAMN09224735
20171793	SAMN11116496
20171810	SAMN09224234
20171860	SAMN09224706
20171873	SAMN09218778
20171904	SAMN09214303
20171911	SAMN09219234
20171960	SAMN09224738
20172200	SAMN09224123
20172220	SAMN09219189
20172462	SAMN09224238
20172479	SAMN11116561
20172521	SAMN11116569
20172523	SAMN11116571
20172524	SAMN11116572
20172529	SAMN11116577
20172568	SAMN09224506
20172804	SAMN09219427
20172842	SAMN09218508
20172850	SAMN09218794
20172941	SAMN09224646
20173014	SAMN11116681
20173081	SAMN11116685
20173375	SAMN09218987
20173418	SAMN11116806
20173651	SAMN09224126
20173897	SAMN11116946
20173947	SAMN11116981
20173959	SAMN11116992

24--27--414	14	No	1
24--27--422	14	No	1
24--27--424	14	No	1
24--27--425	14	No	1
24--27--428	14	No	1
24--27--431	14	No	1
24--27--432	14	No	1
24--27--433	14	No	1
24--27--437	14	No	1
24--27--480	14	No	1
24--27--488	14	No	1
24--27--519	14	No	1
24--27--653	14	No	1
24--27--703	14	No	1
24--27--729	14	No	1
24--27--925	14	No	1
24--27--932	14	No	1
24--309--629	14	No	1
24--309--8	14	No	1
24--319--551	14	No	1
24--362--47	14	No	1
24--53--117	14	No	1
24--53--551	14	No	1
24--7--36	14	No	1
34--309--551	14	No	1
427--27--445	14	No	1
24--0--28	14	Yes	1
24--4--28	14	Yes	1

20174187	SAMN11116997
20174231	SAMN11117036
20174349	SAMN11117048
20174462	SAMN11117115
20174489	SAMN11117138
20174606	SAMN11117217
20174951	SAMN11117259
20174952	SAMN11117260
20174976	SAMN11117282
20175141	SAMN11117288
20175190	SAMN11117331
20175198	SAMN11117339
20175200	SAMN11117341
20175467	SAMN11117391
20175663	SAMN11117444
20175665	SAMN11117446
20175710	SAMN11120839
20175754	SAMN11120879
20175797	SAMN11120916
20175961	SAMN21546510
20176120	SAMN11121013
20176516	SAMN11121122
20176821	SAMN11121220
20176824	SAMN11121223
20176847	SAMN11121245
20177076	SAMN11121322
20177127	SAMN11121360
20177141	SAMN11121370
20177143	SAMN11121372
20180275	SAMN11121384
20180284	SAMN11121392
20180418	SAMN11121477
20180450	SAMN09231324
20180494	SAMN11121505
20180556	SAMN11121527
20181171	SAMN11121538
20181176	SAMN11121543
20181178	SAMN11121545
20181197	SAMN11121563
20181206	SAMN11121572
20181348	SAMN11121664
20181352	SAMN11121668
20181376	SAMN11121689
20181380	SAMN11121693
20181450	SAMN11121752
20181637	SAMN11122320
20181801	SAMN11122334

20182481	SAMN11122444
20182508	SAMN11122470
20182716	SAMN11122540
20182725	SAMN11122548
20182751	SAMN14150465
20182752	SAMN14150466
20182781	SAMN14150490
20182805	SAMN14150502
20182825	SAMN11122583
20182842	SAMN11122598
20183166	SAMN14150594
20183174	SAMN14150602
20183210	SAMN21546511
20183230	SAMN21546512
20183280	SAMN14150667
20183320	SAMN14150703
20183531	SAMN14150723
20183566	SAMN11122752
20183573	SAMN11122757
20183605	SAMN14150754
20183610	SAMN14150759
20183621	SAMN14150768
20183672	SAMN14150811
20183690	SAMN14150828
20184219	SAMN14150893
20184237	SAMN14150911
20184249	SAMN14150922
20184252	SAMN14150925
20184396	SAMN14150992
20184421	SAMN14151009
20184439	SAMN11122773
20184476	SAMN11122809
20184493	SAMN11122825
20184505	SAMN11122836
20184506	SAMN11122837
20184553	SAMN14151025
20184564	SAMN14151036
20184601	SAMN14151060
20184665	SAMN14151121
20184677	SAMN14151125
20184691	SAMN14151139
20184711	SAMN14151158
20184751	SAMN14151192
20184765	SAMN14151206
20184787	SAMN14151228
20184805	SAMN14151246
20184861	SAMN14151298

20184862	SAMN14151299
20184865	SAMN14151302
20184876	SAMN11122885
20184886	SAMN11122895
20184912	SAMN11122920
20184925	SAMN14151319
20184952	SAMN21546513
20185002	SAMN14151333
20185031	SAMN14151360
20185053	SAMN14157723
20185060	SAMN14157730
20185089	SAMN14157756
20185098	SAMN14157765
20185128	SAMN14157794
20185136	SAMN14157802
20185141	SAMN14157807
20185221	SAMN11123015
20185237	SAMN14157842
20185288	SAMN14157890
20185418	SAMN14157921
20185513	SAMN14157993
20185661	SAMN14158034
20185670	SAMN14158043
20185678	SAMN14158051
20185895	SAMN14158110
20185969	SAMN14158138
20185977	SAMN14158144
20186006	SAMN14158173
20186011	SAMN14158178
20186014	SAMN14158181
20186015	SAMN14158182
20186261	SAMN14158264
20186265	SAMN14158268
20186288	SAMN14158287
20186339	SAMN14158330
20186367	SAMN21546514
20190069	SAMN14158368
20190313	SAMN14158384
20190342	SAMN14158412
20190367	SAMN14158437
20190409	SAMN14158456
20191504	SAMN14158548
20191555	SAMN14158593
20191557	SAMN14158595
20191619	SAMN14158653
20191648	SAMN14158681
20191670	SAMN14158703

20191682	SAMN14158715
20192232	SAMN14168680
20192254	SAMN14168702
20193961	SAMN14168743
20193966	SAMN21546515
20194215	SAMN21546516
20194485	SAMN20331822
20194524	SAMN14168833
20194555	SAMN14168864
20194587	SAMN20331840
20194589	SAMN20331842
20194593	SAMN20331846
20194630	SAMN14168897
20194640	SAMN14168907
20194643	SAMN14168910
20194655	SAMN14168922
20194668	SAMN14168934
20194674	SAMN14168940
20194675	SAMN14168941
20194679	SAMN14168945
20194685	SAMN14168951
20194708	SAMN14168974
20194709	SAMN14168975
20194726	SAMN14168992
20194747	SAMN14169011
20195605	SAMN21546517
20195608	SAMN21546518
20195615	SAMN20331911
20195890	SAMN20332002
20195919	SAMN20332023
20196075	SAMN14169087
20196186	SAMN20332095
20196336	SAMN20332184
20196757	SAMN14169113
20196822	SAMN14169175
20196827	SAMN14169180
20196891	SAMN14169212
20196902	SAMN14169223
20196908	SAMN14169227
20198057	SAMN14169253
20198585	SAMN14169301
2008228002	ERR742404
2009211239	ERR433636
2009212860	ERR742636
2009214044	ERR662365
2009214582	ERR742348
2009218323	ERR1422682

2009218327	ERR600092
2009218339	ERR742657
2010205239	ERR1202584
2010205298	ERR505735
2010209967	ERR1202594
2010220239	ERR662366
2012210256	ERR662389
2012214505	ERR662404
2012220641	ERR662322
2013201224	ERR600180
2013206201	ERR600173
2013211307	ERR596405
0104-99	ERR433784
2232148_3870181_6200	ERR311090
2896-99	ERR460191
3130-06	ERR600305
3751-02	ERR600297
4066-00	ERR1422637
5236301_3328974_679	ERR516664
5447983_3404170_1195	ERR311100
5541578_3454925_1644	ERR311140
5552702_3484214_1943	ERR316809
5554951_3476338_1800	ERR311123
6336-00	ERR425413
B22581	ERR316646
C6640	ERR501493
D26870	ERR316580
D51814	ERR316599
D9236	ERR316654
GPS_AR_14297	ERR2667719
GPS_AR_14326	ERR2667728
GPS_AR_15217	ERR2667775
GPS_AR_15226	ERR2667778
GPS_AR_15271	ERR2667785
GPS_AR_15322	ERR2667798
GPS_AR_15388	ERR2667812
GPS_AR_16000	ERR2667831
GPS_AR_16096	ERR2667854
GPS_AR_16187	ERR2667866
GPS_AR_16195	ERR2667868
GPS_AR_16387	ERR2667916
GPS_AR_16424	ERR2667924
GPS_AR_17339	ERR2667976
GPS_AR_17405	ERR2667985
GPS_AR_17564	ERR2668003
GPS_BD_1031801	ERR2225355
GPS_BD_10863	ERR2225294

GPS_BD_1100102305	ERR2213046
GPS_BD_1105260100	ERR2212976
GPS_BD_11720	ERR2213029
GPS_BD_30001	ERR2225390
GPS_BD_32725	ERR2212872
GPS_BD_33177	ERR2212871
GPS_BD_4101072113	ERR2225373
GPS_BD_41867	ERR2213023
GPS_BD_43346	ERR2213054
GPS_BD_50196	ERR2212881
GPS_BD_70438	ERR2225270
GPS_BD_71141	ERR2212961
GPS_BD_72527	ERR2212873
GPS_BD_73562	ERR2212819
GPS_BD_74029	ERR2212802
GPS_BD_80003	ERR2213075
GPS_BD_8002	ERR2212916
GPS_BD_9931	ERR2212857
GPS_BR_0030_13	ERR2089455
GPS_CH_GZ13115	ERR2352137
GPS_GH_92	ERR1788325
GPS_GM_0183	ERR714192
GPS_GM_0192	ERR714201
GPS_GM_0257	ERR714266
GPS_GM_0343	ERR714182
GPS_GM_0426	ERR714350
GPS_GM_0468	ERR714392
GPS_GM_0489	ERR714413
GPS_GM_0504	ERR714428
GPS_GM_0515	ERR714439
GPS_GM_0520	ERR730142
GPS_GM_0556	ERR730178
GPS_GM_0623	ERR730243
GPS_GM_0645	ERR730265
GPS_GM_0659	ERR730279
GPS_GM_0719_R1	ERR913250
GPS_GM_0728	ERR730348
GPS_GM_0737	ERR730357
GPS_GM_0748	ERR730368
GPS_GM_0749	ERR730369
GPS_GM_0759	ERR730379
GPS_GM_0761	ERR730381
GPS_GM_0762	ERR730382
GPS_GM_0765	ERR730385
GPS_GM_0767	ERR730387
GPS_GM_0770	ERR730390
GPS_GM_0792	ERR730412

GPS_GM_0794	ERR730414
GPS_GM_0796	ERR730416
GPS_GM_0814	ERR730434
GPS_GM_0816	ERR730436
GPS_GM_0836	ERR730456
GPS_GM_0845_R1	ERR913248
GPS_GM_0955	ERR870078
GPS_GM_0970	ERR870090
GPS_GM_1036	ERR870153
GPS_GM_1066	ERR870182
GPS_GM_1083	ERR870197
GPS_GM_1122	ERR870227
GPS_GM_1155	ERR870260
GPS_GM_1164	ERR870269
GPS_GM_1166	ERR870271
GPS_GM_1271_R1	ERR2089239
GPS_GM_1276	ERR913193
GPS_GM_1431	ERR913333
GPS_GM_1497	ERR913377
GPS_GM_1624	ERR997568
GPS_GM_1637	ERR997580
GPS_GM_1641	ERR997584
GPS_GM_1653	ERR997596
GPS_GM_1724	ERR997660
GPS_GM_1910	ERR1106807
GPS_GM_1956	ERR1191948
GPS_GM_1979	ERR1191971
GPS_GM_1988	ERR1333960
GPS_GM_2005	ERR1191996
GPS_GM_2047	ERR1192025
GPS_GM_2059	ERR1192037
GPS_GM_2096	ERR1191849
GPS_GM_2166	ERR1192119
GPS_GM_2197	ERR1430806
GPS_GM_2276	ERR1430890
GPS_GM_2336	ERR1430950
GPS_GM_2725	ERR1658106
GPS_GM_2730	ERR1658111
GPS_GM_2818	ERR1658195
GPS_GM_2831	ERR1658207
GPS_GM_2863	ERR1658233
GPS_GM_2866	ERR1658236
GPS_GM_2972	ERR2089105
GPS_GM_2984	ERR2089117
GPS_GM_3061	ERR2089193
GPS_GM_3249	ERR2089949
GPS_GM_3269	ERR2089969

GPS_GM_3275	ERR2089988
GPS_HK_302	ERR1453630
GPS_HK_316	ERR1453644
GPS_HK_317	ERR1453645
GPS_HK_342	ERR1453669
GPS_HK_349	ERR1453676
GPS_HK_357	ERR1453684
GPS_HK_363	ERR1453690
GPS_HK_371	ERR1453698
GPS_HK_373	ERR1453700
GPS_HK_375	ERR1453702
GPS_HK_409	ERR1453736
GPS_HK_414	ERR1453741
GPS_HK_421	ERR1453748
GPS_HK_423	ERR1453750
GPS_HK_429	ERR1453756
GPS_HK_46	ERR1214528
GPS_HK_470	ERR2667092
GPS_HK_493	ERR2667112
GPS_IL_196678	ERR2089840
GPS_IL_24116	ERR2089784
GPS_IL_24157	ERR2090549
GPS_IL_25903	ERR2091214
GPS_IL_26505	ERR2091279
GPS_IL_26506	ERR2090725
GPS_IL_26652	ERR2091292
GPS_IL_26820	ERR2090735
GPS_IL_26898	ERR2090666
GPS_IL_27026	ERR2090747
GPS_IL_27193	ERR2090764
GPS_IL_27298	ERR2090772
GPS_IL_27628	ERR2090862
GPS_IL_27666	ERR2090831
GPS_IL_27750	ERR2090868
GPS_IL_9345	ERR2091010
GPS_IN_B1018	ERR3655161
GPS_IN_B1908	ERR3655184
GPS_IN_B2994	ERR2668046
GPS_IN_B4756	ERR3655141
GPS_IN_B8022	ERR3655434
GPS_IN_BP1737	ERR2668036
GPS_IN_BP1823	ERR2667221
GPS_IN_BP388	ERR3655418
GPS_IN_C111	ERR2667237
GPS_IN_C126_1	ERR2667258
GPS_IN_C13	ERR2667252
GPS_IN_C59_2	ERR2667256

GPS_IN_CMC_1	ERR3899352
GPS_IN_CMC_105	ERR3899450
GPS_IN_CMC_16	ERR3899476
GPS_IN_CMC_17	ERR3899479
GPS_IN_CMC_20	ERR3899488
GPS_IN_CMC_24	ERR3899497
GPS_IN_CMC_4	ERR3899439
GPS_IN_CMC_43	ERR3899216
GPS_IN_CMC_86	ERR3899295
GPS_IN_CMC_92	ERR3899431
GPS_IN_CNBC1124	ERR2668033
GPS_IN_CS08	ERR3655125
GPS_IN_CS1050	ERR2667229
GPS_IN_CS138	ERR2668026
GPS_IN_CS139	ERR2668039
GPS_IN_CS1487	ERR3655168
GPS_IN_CS164	ERR2667225
GPS_IN_CS215	ERR2668045
GPS_IN_G100	ERR3227774
GPS_IN_G127	ERR3227778
GPS_IN_G137	ERR3227759
GPS_IN_G21	ERR3227823
GPS_IN_G57	ERR2090150
GPS_IN_G58	ERR2090156
GPS_IN_G77	ERR2090129
GPS_IN_IIT_5	ERR2090196
GPS_IN_KMC2770928	ERR2668034
GPS_IN_P02	ERR1638454
GPS_IN_P05	ERR1638457
GPS_IN_P15	ERR1756418
GPS_IN_P2004	ERR3227784
GPS_IN_P504	ERR2090227
GPS_IN_P56	ERR1756436
GPS_IN_P75	ERR1756391
GPS_IN_P86	ERR1756478
GPS_IN_PR_D78	ERR2090296
GPS_IN_PR_H165	ERR2090314
GPS_IN_PR_M149	ERR2090378
GPS_IN_SF48508	ERR3227773
GPS_IN_TVM_3	ERR2668027
GPS_KE_2009818766	ERR3217454
GPS_KE_2009818938	ERR3217506
GPS_KE_2010831697	ERR3192339
GPS_KE_2010832074	ERR3192255
GPS_KE_2011743327	ERR3192365
GPS_KE_2011748556	ERR3192390
GPS_KE_2017CS0038	ERR3192311

GPS_KE_2017CS0366	ERR3164327
GPS_KE_2017CS0424	ERR3164457
GPS_KE_2017CS0425	ERR3655147
GPS_KH_COMRU010_R1	ERR3899252
GPS_KH_COMRU11	ERR2680564
GPS_KH_COMRU111	ERR2680661
GPS_KH_COMRU145	ERR2680695
GPS_KH_COMRU185	ERR2680735
GPS_KH_COMRU23	ERR2680576
GPS_KH_COMRU256_R1	ERR3899398
GPS_KH_COMRU269	ERR2680818
GPS_KH_COMRU293	ERR2681399
GPS_KH_COMRU301	ERR2680848
GPS_KH_COMRU324	ERR2680870
GPS_KH_COMRU341	ERR2680886
GPS_KH_COMRU367	ERR2680935
GPS_KH_COMRU371	ERR2680939
GPS_KH_COMRU376	ERR2680944
GPS_KH_COMRU38	ERR2680591
GPS_KH_COMRU381	ERR2680949
GPS_KH_COMRU382	ERR2680950
GPS_KH_COMRU390	ERR2680958
GPS_KH_COMRU391	ERR2680959
GPS_KH_COMRU403	ERR2680971
GPS_KH_COMRU412	ERR2680979
GPS_KH_COMRU436	ERR2681003
GPS_KH_COMRU472	ERR2681038
GPS_KH_COMRU542	ERR2681108
GPS_KH_COMRU554	ERR2681120
GPS_KH_COMRU566	ERR2681132
GPS_KH_COMRU570	ERR2681136
GPS_KH_COMRU572	ERR2681138
GPS_KH_COMRU573	ERR2681139
GPS_KH_COMRU604	ERR2681170
GPS_KH_COMRU609	ERR2681175
GPS_KH_COMRU614	ERR2681180
GPS_KH_COMRU628	ERR2681194
GPS_KH_COMRU63	ERR2680616
GPS_KH_COMRU671	ERR2681235
GPS_KH_COMRU674	ERR2681238
GPS_KH_COMRU679	ERR2681242
GPS_KH_COMRU7	ERR2680560
GPS_KH_COMRU713	ERR2681274
GPS_KH_COMRU715	ERR2681276
GPS_KH_COMRU726	ERR2681287
GPS_KH_COMRU757	ERR2681317
GPS_KH_COMRU760	ERR2681320

GPS_KH_COMRU776	ERR2681336
GPS_KH_COMRU780	ERR2681340
GPS_KH_COMRU781	ERR2681341
GPS_KH_COMRU789	ERR2681349
GPS_KH_COMRU804	ERR2681364
GPS_KH_COMRU830	ERR2681390
GPS_KH_COMRU835	ERR2681395
GPS_KH_COMRU841	ERR2680912
GPS_KH_COMRU861	ERR2612114
GPS_KH_COMRU902	ERR2667410
GPS_KH_COMRU904	ERR2667412
GPS_KH_COMRU922	ERR2667430
GPS_KH_COMRU924	ERR2667432
GPS_KH_COMRU951	ERR2667459
GPS_KH_COMRU97	ERR2680648
GPS_KH_COMRU980	ERR2667488
GPS_MW_1024164_R1	ERR1202379
GPS_MW_1199	ERR862461
GPS_MW_1368	ERR862467
GPS_MW_1410	ERR869906
GPS_MW_1412_R1	ERR774623
GPS_MW_154984	ERR862543
GPS_MW_154985	ERR869859
GPS_MW_1615	ERR862448
GPS_MW_1653	ERR869852
GPS_MW_18759	ERR869954
GPS_MW_19045	ERR869902
GPS_MW_2581_R1	ERR774614
GPS_MW_BND1DC	ERR1191899
GPS_MW_BQN1QT	ERR1202130
GPS_MW_C12111	ERR1202354
GPS_MW_C3713	ERR1202477
GPS_MW_C7561	ERR1202285
GPS_MW_C9873_R1	ERR774598
GPS_MW_D24847	ERR1202278
GPS_MW_D28100	ERR1202252
GPS_MW_D39268_R1	ERR862267
GPS_MW_D44857	ERR1202361
GPS_NG_030190327	ERR3899249
GPS_NG_030200272	ERR3899318
GPS_NG_20130469	ERR2089638
GPS_NG_30030585	ERR2089630
GPS_NG_HB3633	ERR2090504
GPS_NG_HB3946	ERR2090486
GPS_NG_HBB1379	ERR2089787
GPS_NG_HBB1640	ERR2089596
GPS_NG_M2624	ERR2090466

GPS_NG_MM2642	ERR2090476
GPS_NG_MMB1383	ERR2089589
GPS_NP_0016	ERR980213
GPS_NP_0022	ERR980218
GPS_NP_0031	ERR979884
GPS_NP_0032	ERR979885
GPS_NP_0033	ERR979886
GPS_NP_0054	ERR979906
GPS_NP_2097	ERR979953
GPS_NP_2489	ERR980002
GPS_NP_4041	ERR980030
GPS_NP_4047	ERR980033
GPS_NP_4088	ERR980050
GPS_NP_4110	ERR980059
GPS_NP_4144	ERR980073
GPS_NP_4145	ERR980074
GPS_NP_6027	ERR980090
GPS_NP_6036	ERR980092
GPS_NP_6107	ERR980101
GPS_NP_6169	ERR980112
GPS_NP_6181	ERR980113
GPS_NP_6362	ERR980130
GPS_NP_6531	ERR980150
GPS_NP_6837	ERR980190
GPS_PG_UA_1246	ERR2668110
GPS_PK_007	ERR1799705
GPS_PK_100	ERR1795517
GPS_TG_P0897	ERR1106700
GPS_TG_P1462	ERR1106710
GPS_TH_07B00729	ERR1202598
GPS_TH_12B00648	ERR1333968
GPS_TH_12B02382	ERR1202611
GPS_TH_13B03011	ERR1202612
GPS_TH_13B05245_R1	ERR1202614
GPS_TH_13B05246_R1	ERR3899235
GPS_TH_14B07344_R1	ERR3899248
GPS_TH_BK_0256	ERR884479
GPS_TH_BK_0365	ERR884515
GPS_TH_BK_0410	ERR884531
GPS_TH_BK_0417	ERR884534
GPS_TH_BK_0489	ERR884557
GPS_TH_BK_0509	ERR884568
GPS_TH_BK_0525	ERR884580
GPS_TH_BK_0528	ERR884582
GPS_TH_BK_0547	ERR884594
GPS_TH_BK_0555	ERR884600
GPS_TH_BK_0587	ERR884611

GPS_TH_BK_0594	ERR884616
GPS_TH_BK_0662	ERR884650
GPS_TH_BK_0668	ERR884653
GPS_TH_BK_0670	ERR884655
GPS_TH_BK_0680	ERR884663
GPS_TH_BK_0684	ERR884665
GPS_TH_BK_0722	ERR884688
GPS_US_PATH354	ERR742676
GPS_US_PATH3606	ERR750810
GPS_US_PATH362	ERR742681
GPS_US_PATH3621	ERR750823
GPS_US_PATH3632	ERR750833
GPS_US_PATH3637	ERR750838
GPS_US_PATH3639	ERR750840
GPS_US_PATH3664	ERR750861
GPS_US_PATH3680	ERR750865
GPS_US_PATH3756	ERR750875
GPS_US_PATH3758	ERR750877
GPS_US_PATH3761	ERR1214428
GPS_US_PATH3764	ERR750881
GPS_US_PATH3766	ERR750882
GPS_US_PATH3767	ERR750883
GPS_US_PATH397	ERR1214405
GPS_US_PATH507	ERR742708
GPS_US_PATH515	ERR742713
GPS_US_PATH524	ERR742719
GPS_US_PATH5339	ERR750958
GPS_US_PATH5342	ERR750960
GPS_US_PATH5346	ERR750963
GPS_US_PATH535	ERR742728
GPS_US_PATH544	ERR742735
GPS_US_PATH5449	ERR751005
GPS_US_PATH5450	ERR751006
GPS_US_PATH5509	ERR751043
GPS_US_PATH554	ERR1214423
GPS_US_PATH5561	ERR751092
GPS_US_PATH5582	ERR751118
GPS_US_PATH5590	ERR751125
GPS_US_PATH5595	ERR751130
GPS_US_PATH5611	ERR751143
GPS_US_PATH562	ERR742747
GPS_US_PATH606	ERR742782
GPS_US_PATH620	ERR742791
GPS_US_PATH622	ERR742792
GPS_US_PATH633	ERR742802
GPS_US_PATH652	ERR742817
GPS_US_PATH659	ERR845764

GPS_US_PATH6669	ERR1422349
GPS_US_PATH673	ERR742831
GPS_US_PATH7006	ERR1422390
GPS_US_PATH728	ERR742852
GPS_US_PATH740	ERR742862
GPS_US_PATH747	ERR742867
GPS_US_PATH750	ERR742870
GPS_ZA_1017	ERR600606
GPS_ZA_1054	ERR632983
GPS_ZA_1113	ERR633040
GPS_ZA_1137	ERR633064
GPS_ZA_1140	ERR633067
GPS_ZA_1192	ERR633118
GPS_ZA_1217	ERR646554
GPS_ZA_1253	ERR646590
GPS_ZA_1294	ERR730530
GPS_ZA_1323	ERR730559
GPS_ZA_1335	ERR730571
GPS_ZA_1377	ERR736902
GPS_ZA_1387	ERR701787
GPS_ZA_1412	ERR701812
GPS_ZA_1451	ERR701851
GPS_ZA_1549	ERR714519
GPS_ZA_1656	ERR730754
GPS_ZA_1673	ERR714548
GPS_ZA_1674	ERR714549
GPS_ZA_1703	ERR714578
GPS_ZA_1710	ERR714585
GPS_ZA_1729	ERR714604
GPS_ZA_1785	ERR730788
GPS_ZA_1807	ERR730810
GPS_ZA_1847	ERR730850
GPS_ZA_1953	ERR730861
GPS_ZA_2022	ERR730930
GPS_ZA_2052	ERR736907
GPS_ZA_2115	ERR730643
GPS_ZA_2329	ERR774005
GPS_ZA_2429	ERR774291
GPS_ZA_2723	ERR774205
GPS_ZA_2805	ERR1066195
GPS_ZA_2849	ERR1066249
GPS_ZA_3055	ERR1065414
GPS_ZA_843	ERR600439
GPS_ZA_911	ERR600507
GPS_ZA_carriage_SP111	ERR925052
GPS_ZA_carriage_SP133	ERR925038
GPS_ZA_carriage_SP1552	ERR1065675

GPS_ZA_carriage_SP158	ERR925048
GPS_ZA_carriage_SP1755	ERR1066154
GPS_ZA_carriage_SP1773	ERR1066170
GPS_ZA_carriage_SP1824	ERR1065767
GPS_ZA_carriage_SP1953	ERR1065452
GPS_ZA_carriage_SP216	ERR908860
GPS_ZA_carriage_SP22	ERR900719
GPS_ZA_carriage_SP225	ERR908932
GPS_ZA_carriage_SP235	ERR908917
GPS_ZA_carriage_SP246	ERR908910
GPS_ZA_carriage_SP397	ERR913514
GPS_ZA_carriage_SP417	ERR913486
GPS_ZA_carriage_SP426	ERR913552
GPS_ZA_carriage_SP450	ERR913554
GPS_ZA_carriage_SP536	ERR913064
GPS_ZA_carriage_SP687	ERR909113
GPS_ZA_carriage_SP73	ERR900652
GPS_ZA_carriage_SP747	ERR909118
PI00175	ERR352006
PI0170	ERR433829
PI0179	ERA211402
PI0226	ERA211421
PI0255	ERA211434
PI0259	ERA211403
PNI0145	ERR438993
PNI0194	ERR438997
PNI0305_R1	ERR913251
PNI0438	ERR438995
ZA_GPS_SP10	ERR505900
ZA_GPS_SP17	ERR505907
ZA_GPS_SP209	ERR570445
ZA_GPS_SP215	ERR570451
ZA_GPS_SP297	ERR568485
ZA_GPS_SP319	ERR568508
ZA_GPS_SP372	ERR568561
ZA_GPS_SP458	ERR568647
ZA_GPS_SP527	ERR568711
ZA_GPS_SP558	ERR568742
ZA_GPS_SP600	ERR570412
ZA_GPS_SP75	ERR505965

Supplementary Data 1: *pbp*2x-*dex*B–*ali*A-*pbp*1a sequence

```
CDS             148568..150820
                     /gene="pbpX"
                     /locus_tag="PROKKA_00156"
                     /inference="ab initio prediction:Prodigal:2.60"
                     /inference="similar to AA sequence:UniProtKB:P59676"
                     /codon_start=1
                     /transl_table=11
                     /product="Penicillin-binding protein 2X"
                     /protein_id="Prokka:PROKKA_00156"
                     /translation="MKWTKRVIRYATKNRKSPAENRRRVGKSLSLLSVFVFAIFLVNF
                     AVIIGTGTRFGTDLAKEAKKVHQTTRTVPAKRGTIYDRNGVPIAEDATSYNVYAVIDE
                     NYKSATGKILYVEKTQFNKVAEVFHKYLDMEESYVREQLSQPNLKQVSFGAKGNGITY
                     ANMMSIKKELEAAEVKGIDFTTSPNRSYPNGQFASSFIGLAQLHENEDGSKSLLGTSG
                     MESSLNSILAGTDGIITYEKDRLGNIVPGTEQVSQRTMDGKDVYTTISSLLQSFMETQ
                     MDAFQEKVKGKYMTATLVSAKTGEILATTQRPTFDADTKEGITEDFVWRDILYQSNYE
                     PGSTMKVMMLAAAIDNNTFPGGEVFNSSELKIADATIRDWDVNEGLTGGRMMTFSQGF
                     AHSSNVGMTLLEQKMGDATWLDYLNRFKFGVPTRFGLTDEYAGQLPADNIVNIAQSSF
                     GQGISVTQTQMIRAFTAIANDGVMLEPKFISAIYDPNDQTARKSQKEIVGNPVSKDAA
                     SLTRTNMVLVGTDPVYGTMYNHSTGKPTVTVPGQNVALKSGTAQIADEKNGGYLVGVT
                     DYIFSAVSMSPAENPDFILYVTVQQPEHYSGIQLGEFANPILERASAMKDSLNLQTTA
                     KALEQVSQQSPYPMPSVKDISPGDLAEELRRNLVQPIVVGTGTKIKNSSAEEGKNLAP
                     NQQVLILSDKAEEVPDMYGWTKETAETLAKWLNIELEFQGSGSTVQKQDVRANTAIKD
                     IKKITLTLGD"
     gene            150822..151802
                     /gene="mraY"
                     /locus_tag="PROKKA_00157"
     CDS             150822..151802
                     /gene="mraY"
                     /locus_tag="PROKKA_00157"
                     /EC_number="2.7.8.13"
                     /inference="ab initio prediction:Prodigal:2.60"
                     /inference="similar to AA sequence:UniProtKB:Q2FZ93"
                     /codon_start=1
                     /transl_table=11
                     /product="Phospho-N-acetylmuramoyl-pentapeptide-
                     transferase"
                     /protein_id="Prokka:PROKKA_00157"
                     /translation="MFISISAGIVTFLLTLVGIPAFIQFYRKAQITGQQMHEDVKQHQ
                     AKAGTPTMGGLVFLITSVLVAFFFALFSSQFSNNVGMILFILVLYGLVGFLDDFLKVF
                     RKINEGLNPKQKLALQLLGGIIFYLFYERGGDMLSVFGYQVHLGIFYIVFALFWLVGF
                     SNAVNLTDGVDGLASISVVISLSAYGVIAYMQGQMDILLVILAMIGGLLGFFIFNHKP
                     AKVFMGDVGSLALGGMLAAISMALHQEWTLLIIGIVYVFETTSVMMQVSYFKLTGGKR
                     IFRMTPVHHHFELGGLSGKGNPWSEWKVDFFFWGVGLLASLLTLAILYLM"
     gene            152411..154516
                     /gene="clpC"
                     /locus_tag="PROKKA_00158"
     CDS             152411..154516
                     /gene="clpC"
                     /locus_tag="PROKKA_00158"
                     /inference="ab initio prediction:Prodigal:2.60"
                     /inference="similar to AA sequence:UniProtKB:Q5XCL7"
                     /codon_start=1
                     /transl_table=11
                     /product="putative ATP-dependent Clp protease ATP-binding
                     subunit"
                     /protein_id="Prokka:PROKKA_00158"
                     /translation="MNNNFNNFNNMDDLFNQLMGGMRGYSSENRRYLINGREVTPEEF
                     AHYRATGQLLGNAETDVQMPQQASGMKQDGVLAKLGRNLTAEAREGKLDPVIGRNKEI
```

```
                     QETSEILSRRTKNNPVLVGDAGVGKTAVVEGLAQAIVNGDVPAAIKNKEIISIDISGL
                     EAGTQYRGSFEENVQNLVNEVKEAGNIILFFDEIHQILGAGSTGGDSGSKGLADILKP
                     ALSRGELTVIGATTQDEYRNTILKNAALARRFNEVKVNAPSAENTFKILQGIRDLYQQ
                     HHNVILPDEVLKAAVDYSVQYIPQRSLPDKAIDLVDVTAAHLAAQHPVTDVHAVEREI
                     ETEKDKQEKAVEAEDFEAALNYKTRIAELEKKIENHTEDMKVTASVNDVAESVERMTG
                     IPVSQMGASDIERLKDMAHRLQEKVIGQDKAVEVVARAIRRNRAGFDEGNRPIGSFLF
                     VGSTGVGKTELAKQLALDMFGTQDAIIRLDMSEYSDRTAVSKLIGTTAGYVGYDDNSN
                     TLTERVRRNPYSIILLDEIEKADPQVITLLLQVLDDGRLTDGQGNTVNFKNTVIIATS
                     NAGFGYEANLTEDADKPELMDRLNPFFRPELLNRFNAVIEFSHLTKEDLSKIVDLMLA
                     EVNQTLAKKDIDLVVSQAAKDYITEEGYDEVMGVRPLRRVVEQEIRDKVTDFHLDHLD
                     AKHLEADMEDGVLVIREKA"
     gene            complement(154812..155294)
                     /gene="luxS"
                     /locus_tag="PROKKA_00159"
     CDS             complement(154812..155294)
                     /gene="luxS"
                     /locus_tag="PROKKA_00159"
                     /EC_number="4.4.1.21"
                     /inference="ab initio prediction:Prodigal:2.60"
                     /inference="similar to AA sequence:UniProtKB:Q9RRU8"
                     /codon_start=1
                     /transl_table=11
                     /product="S-ribosylhomocysteine lyase"
                     /protein_id="Prokka:PROKKA_00159"
                     /translation="MSKEVIVESFELDHTIVKAPYVRLIGEETGPKGDIISNYDIRLV
                     QPNEDSIPTAGLHTIEHLLAKLIRTRIDGMIDCSPFGCRTGFHMIMWGRHTSAKIAAV
                     IKDSLKEIAETTTWEDVPGTTIESCGNYKDHSLFSAKEWAKLILEQGISDDAFERHVI
                     "
     gene            complement(155389..156873)
                     /locus_tag="PROKKA_00160"
     CDS             complement(155389..156873)
                     /locus_tag="PROKKA_00160"
                     /inference="ab initio prediction:Prodigal:2.60"
                     /codon_start=1
                     /transl_table=11
                     /product="hypothetical protein"
                     /protein_id="Prokka:PROKKA_00160"
                     /translation="MKKQAFSSEQYLNLQRDHILERINQFDGKLYLEFGGKMLEDFHA
                     ARVLPGYEPDNKIKLLQELKEQVEVVIAINASNIEHSKARGDLGISYDQEVLRLIDKF
                     NELGIFVGSVVITQYAGQPAADAFRNQLEKNGIDSYLHYPIKGYPTDMDHIISPEGMG
                     KNDYIKTSRNLIVVTAPGPGSGKLATCMSNMYHDQINGIKSGYAKFETFPVWNLPLHH
                     PVNLAYEAATADLDDVNMIDPFHLQTYGETTVNYNRDIEIFPVLKRMLERILGKSPYA
                     SPTDMGVNMVGFAITDDEAAVEASKQEIIRRYYQTVLDFKAEKVGEAAVKKIELLMND
                     LGITPADRKVAVVARQKAEETGGPALAFELPNGEIVTGKNSELFGPTAAALINAIKKS
                     ADIAKEVKLIEPEVVKPIQGLKIDHLGSRNPRLHSNEILIALAITATENPDAARAMEE
                     LGNLKGSEAHSTIILTDEDKNVLRKLGINVTFDPYYQYDRLYRK"
     gene            157026..158636
                     /gene="dexB"
                     /locus_tag="PROKKA_00161"
     CDS             157026..158636
                     /gene="dexB"
                     /locus_tag="PROKKA_00161"
                     /EC_number="3.2.1.70"
                     /inference="ab initio prediction:Prodigal:2.60"
                     /inference="similar to AA sequence:UniProtKB:Q99040"
                     /codon_start=1
                     /transl_table=11
                     /product="Glucan 1,6-alpha-glucosidase"
                     /protein_id="Prokka:PROKKA_00161"
                     /translation="MQEKWWHNAVVYQVYPKSFMDSNGDGVGDLPGITSKLDYLAKLG
```

```
                     ITAIWLSPVYDSPMDDNGYDIADYQAIATIFGTMEDMDQLIAEAKKRDIRIIMDLVVN
                     HTSDEHAWFVEACENTDSPERDYYIWRDEPNDLDSIFSGPAWEYDEKSGQYYLHFFSK
                     KQPDLNWENEKLRQKIYEMMNFWIDKGIGGFRMDVIDMIGKIPDEKVVNNGPMLHPYL
                     KEMNQATFGDKDLLTVGETWGATPEIAKLYSDPKGQELSMVFQFEHIGLQYQEGQPKW
                     HYQKELNIAKLKEIFNKWQTELGVEDGWNSLFWNNHDLPRIVSIWGNDQEYREKSAKA
                     FAILLHLMRGTPYIYQGEEIGMTNYPFETLDQVEDIESLNYAREALEKGVPIEEIMDS
                     IRVIGRDNARTPMQWDESKNAGFSTGQPWLAVNPNYEMINVQEALANPDSIFYTYQKL
                     VQIRKENSWLIRADFELLDTVDKVFAYIRKDGDRRFLVVANLSNEEQDLTVEGKVKSV
                     LIENTLAQEVFEKQILVPWDAFCVEMTD"
     gene            complement(158767..160176)
                     /locus_tag="PROKKA_00162"
     CDS             complement(158767..160176)
                     /locus_tag="PROKKA_00162"
                     /inference="ab initio prediction:Prodigal:2.60"
                     /inference="protein motif:Pfam:PF00665.20"
                     /codon_start=1
                     /transl_table=11
                     /product="Integrase core domain protein(tranposase)"
                     /protein_id="Prokka:PROKKA_00162"
                     /translation="MNETKKYLVIKTIAQGKKTKKRACVELNLSERQINRLLLAYQQK
                     GKEAFRHGNRNRKPKHAIPDEIKERILKKYLSYETYKPNVLHFCELLAEEEEISLSDT
                     TVRKILYKENILSPKSHRKTKKRIRKQAKLNLNQSLDNPILPTAKDFLEDPKKVHPSR
                     PRKKFAGELIQMDASPHAWFGPETTNLHLAIDDASGNILGAYFDKQETLNAYYHVLEQ
                     ILANHGIPLQMKTDKRTVFTYQASNSKKMEDDTYTQFGYACHQLGILLETTSIPQAKG
                     RVERLNQTLQSRLPIELERNKIHTLEEANTFLLSYIQTFNEQFGNKTKLSVFEEAPNP
                     SERNLILARLAERVVDSGHHIRFQNRYYIPTEQGKEVYFIRKTKALVIKAFDGDIYLN
                     IADKIYHTKELLDHELYSKNFEQEPEQKKKDASISLHKPIRGNSHLSNNTFIKIKRIM
                     KSLLVRSLILLNYKYSKMK"
     gene            160350..162332
                     /gene="sarA"
                     /locus_tag="PROKKA_00163"
     CDS             160350..162332
                     /gene="aliA"
                     /locus_tag="PROKKA_00163"
                     /inference="ab initio prediction:Prodigal:2.60"
                     /inference="similar to AA sequence:UniProtKB:P31306"
                     /codon_start=1
                     /transl_table=11
                     /product="76 kDa cell surface lipoprotein"
                     /protein_id="Prokka:PROKKA_00163"
                     /translation="MKSSRLFALAGVTLLAATTLAACSGSGSSTKGEKTFSYIYETDP
                     DNLNYLTTGKTATANITSNVIDGLLENDRYGNFVPSMAEDWSVSKDGLTYTYTIRKDA
                     KWYTSEGEEYAAVKAQDFVTGLKYAADKKSDALYLVQESIKGLDAYVKGEIKDFSQVG
                     IKALDDQTVQYTLNKPESFWNSKTTMGVLAPVNEEFLNSKGDDFAKATDPSSLLYNGP
                     YLLKSIVTKSSVEFAKNPNYWDKDNVHVDKVKLSFWDGQDTSKPAENFKDGSLTAARL
                     YPTSSSFAELEKSMKDNIVYTQQDSITYLVGTNIDRQSYKYTSKTSDEQKASTKKALL
                     NKDFRQAIAFGFDRTAYAYQLNGQTGASKILRNLFVPPTFVQADGKNFGDMVKEKLVT
                     YGDEWKDVNLADSQDGLYNPEKANAEFAKAKSALQAEGVQFPIHLDMPVDQTATTKVQ
                     RVQSMKQSLEATLGADNVIIDIQQLQKDEVNNITYFAENAAGEDWDLSDNVGWGPDFA
                     DPSTYLDIIKPSVGESTKTYLGFDSGEDNVAAKKVGLYDYEKLVTEAGDEATDVAKRY
                     DKYAAAQAWLTDSALIIPTTSRTGRPILSKIVPFTIPFALSGNKGTSEPVLYKYLELQ
                     DKAVTVDEYQKAQEKWMKEKEESNKKAQEDLAKHVK"
     gene            162632..167935
                     /locus_tag="PROKKA_00164"
     CDS             162632..167935
                     /locus_tag="PROKKA_00164"
                     /EC_number="3.2.1.97"
                     /inference="ab initio prediction:Prodigal:2.60"
                     /inference="similar to AA sequence:UniProtKB:Q8DR60"
                     /codon_start=1
```

/transl_table=11
/product="Endo-alpha-N-acetylgalactosaminidase precursor"
/protein_id="Prokka:PROKKA_00164"
/translation="MNKGLFEKRCKYSIRKFSLGVASVMIGAAFFGTSPVLADSVQSG
STANLPADLATALAIAKENDGRDFEAPKVGEDQGSPEVTDGPKTEEELLALEKEKPAE
EKPKEDKPAAAKPETPKTVTPEWQTVEKKEQKGTVTIREEKGVRYNQLSSTAQNDNAG
KPALFEKKGLTVDANGNATVDLTFKEDSEKGKSRFGVFLKFKDTKNNVFVGYDKDGWF
WEYKIPGNSTWYKGNRVAAPETGSINRLSITLKSDGQLNASNNDVNLFDTVTLPAAVN
DHLKNEKKILLKAGSYDDERTVVSVKTDNQEGVKTEDTPAEKETGPEVDDSKVTYDTI
QSKVLKAVIDQAFPRVKEYSLNGHTLPGQVQQFNQVFINNHRITPEVTYKKINETTAE
YLMKLRDDAHLINAEMTVRLQVVDNQLHFDVTKIVNHNQVTPGQKIDDERKLLSSISF
LGNALVSVSSDQTGAKFDGATMSNNTHVSGDDHIDVTNPMKDLAKGYMYGFVSTDKLA
AGVWSNSQNSYGGGSNDWTRLTAYKETVGNANYVGIHSSEWQWEKAYKGIVFPEYTKE
LPSAKVVITEDANADKKVDWQDGAIAYRSIMNNPQGWEKVKDITAYRIAMNFGSQAQN
PFLMTLDGIKKINLHTDGLGQGVLLKGYGSEGHDSGHLNYADIGKRIGGVEDFKTLIE
KAKKYGAHLGIHVNASETYPESKYFNEKILRKNPDGSYNYGWNWLDQGINIDAAYDLA
HGRLARWEDLKKKLGDGLDFIYVDVWGNGQSGDNGAWATHVLAKEINKQGWRFAIEWG
HGGEYDSTFHHWAADLTYGGYTNKGINSAITRFIRNHQKDAWVGDYRSYGGAANYPLL
GGYSMKDFEGWQGRSDYNGYVTNLFAHDVMTKYFQHFTVSKWENGTPVTMTDNGSTYK
WTPEMRVELVDADNNKVVVTRKSNDVNSPQYRERTVMLNGRVIQDGSAYLTPWNWDAN
GKKLSTDKEKMYYFNTQAGATTWTLPSDWAKSKVYLYKLTDQGKTEEQELTVKDGKIT
LDLLANQPYVLYRSKQTNPEMSWSEGMHIYDQGFNSGTLKHWTISGDASKADIVKSQG
ANDMLRIQGNKEKVSLTQKLTGLKPNTKYAVYVGVDNRSNAKASITVNTGEKEVTTYT
NKSLALNYVKAYAHNTRRNNATVDDTSYFQNMYAFFTTGSDVSNVTLTLSREAGDQAT
YFDEIRTFENNSSMYGDKHDTGQGTFKQDFENVAQGIFPFVVGGVEGVEDNRTHLSEK
HDPYTQRGWNGKKVDDVIEGNWSLKTNGLVSRRNLVYQTIPQNFRFEAGKTYRVTFEY
EAGSDNTYAFVVGKGEFQSGRRGTQASNLEMHELPNTWTDSKKAKKATFLVTGAETGD
TWVGIYSTGNVSNTRGDSGGNANFRGYNDFMMDNLQIEEITLTGKMLTENALKNYLPT
VAMTNYTKESMDALKEAVFNLSQADDDISVEEAHAEIAKIEALKNALVQKKTALVADD
FASLDAPAQPGEGLENAFDGNVSSLWHTSWNGGDVGKPTTMVLKEPTEITGLRYVPRG
SGSNGNLRDVKLVVTDESGKEHTFTATDWPDNNKPKDIDFGKTIKAKQIVLTGTKTYG
DGGDKYQSAAELIFTRPQIAETPLDLSGYEAALAKAQKLTDKDNQEEVASVQASMKYA
TDNHLLTERMVEYFADYLNQLKDSATKPDAPTVEKPEFKLSSLASDQGKTPDYKQEID
RPETPEQILPATGESQSDTALFLAGVSLALSALFVVKTKKD"
gene complement(168102..170261)
/gene="ponA"
/locus_tag="PROKKA_00165"
CDS complement(168102..170261)
/gene="ponA"
/locus_tag="PROKKA_00165"
/inference="ab initio prediction:Prodigal:2.60"
/inference="similar to AA sequence:UniProtKB:Q04707"
/codon_start=1
/transl_table=11
/product="**Penicillin-binding protein 1A**"
/protein_id="Prokka:PROKKA_00165"
/translation="MKKPTILRLLKYLSISFLSLVIAAIVLGGGVFFYYVSKAPSLSE
SKLVATTSSKIYDNKNQLIADLGSERRVNAQANDIPTNLVKAIVSIEDHRFFDHRGID
TIRILGAFLRNLQSNSLQGGSTLTQQLIKLTYFSTSTSDQTISRKAQEAWLAIQLEQK
ATKQEILTYYINKVYMSNGNYGMQTAAQNYYGKDLNNLSLPQLALLAGMPQAPNQYDP
YSHPKAAQDRRNLVLSEMKNQGYISAEQYEKAVNTPITDGLQSLKSASNYPAYMDNYL
KEVINQVEEETGYNLLTTGMDVYTNVDQEAQKHLWDIYNTDEYVAYPDDELQVASTIV
DVSNGKVIAQLGARHQSSNVSFGINQAVETNRDWGSTMKPITDYAPALEYGVYDSTAT
IVHDEPYNYPGTDIPVYNWDRGYFGNITLQYALQQSRNVPAVETLNKVGLNRAKTFLN
GLGIDYPSIHYSNAISSNTTESNKKYGASSEKMAAAYAAFANGGTYYKPMYIHKVVFS
DGSEKEFSNVGTRAMKETTAYMMTDMMKTVLTYGTGRNAYLAWLPQAGKTGTSNYTDE
EIENHIKTSQFVAPDELFAGYTRKYSMAVWTGYSNRLTPLVGNGLTVAAKVYRSMMTY
LSEGSNPEDWNIPEGLYRNGEFVFKNGARSTWSSPAPQQPPSTESSSSSSDSSTSQSS
STTPSTNNSTTTNPNNNTQQSNTTPDQQNQNPQPAQP"

Supplementary Data 2: *pbp*2x-*dex*B–*ali*A-*pbp*1a sequence

```
CDS                      108485..110737
                         /gene="pbpX"
                         /locus_tag="SAMEA3381655_00710"
                         /inference="ab initio prediction:Prodigal:2.60"
                         /inference="similar to AA
sequence:RefSeq:YP_003878580.1"
                         /codon_start=1
                         /transl_table=11
                         /product="penicillin-binding protein 2X"
                         /protein_id="VRO31244.1"
/translation="MKWTKKVIRYATKNRKSPAENRRRVGKSLSLLSVFVFAIFLVNF
AVIIGTGTRFGTDLAKEAKKVHQTTRTVPAKRGTIYDRNGVPIAEDATSYNVYAVIDE
NYKSATGKILYVEKTQFNKVAEVFHKYLDMEESYVREQLSQPNLKQVSFGAKGNGITY
ANMMSIKKELEAAEVKGIDFATSPNRSYPNGQFASSFIGLAQLHENEDGSKSLLGISG
MESSLNSILAGTDGIITYEKDRLGNIVPGTEQVSQRTMDGKDVYTTISSPLQSFMETQ
MDAFQEKVKGKYMTATLVSAKTGEILATTQRPTFDADTKEGITEDFVWRDILYQSNYE
PGSTMKVMMLAAAIDNNTFPGGEVFNSSELKIADATIRDWDVNEGLTGGRMMTFSQGF
AHSSNVGMTLLEQKMGDATWLDYLNRFKFGVPTRFGLTDEYAGQLPADNIVNIAQSSF
GQGISVTQTQMIRAFTAIANDGVMLEPKFISAIYDPNDQTARKSQKEIVGNPVSKDAA
SLTRTNMVLVGTDPVYGTMYNHSTGKPTVTVPGQNVALKSGTAQIADEKNGGYLVGLT
NYIFSAVSMSPAENPDFILYVTVQQPEHYSGIQLGEFANPILERASAMKDSLNLQTTA
KALEQVSQQSPYPMPSVKDISPGDLAEELRRNLVQPIVVGTGTKIKNSSAEEGKNLAP
NQQVLILSDKVEEVPDMYGWTKETAEIFAKWLNIELEFQGSGSTVQKQDVRANTAIKD
IKKITLTLGD"
     gene                110739..111719
                         /gene="mraY"
                         /locus_tag="SAMEA3381655_00711"
     CDS                 110739..111719
                         /gene="mraY"
                         /locus_tag="SAMEA3381655_00711"
                         /EC_number="2.7.8.13"
                         /inference="ab initio prediction:Prodigal:2.60"
                         /inference="similar to AA
sequence:RefSeq:YP_003446865.1"
                         /codon_start=1
                         /transl_table=11
                         /product="UDP-phosphate-UDP-MurNAc-pentapeptide
                         phospho-MurNAc-pentapeptide transferase"
                         /protein_id="VRO31247.1"
/translation="MFISISAGIVTFLLTLVGIPAFIQFYRKAQITGQQMHEDVKQHQ
AKAGTPTMGGLVFLITSVLVAFFFALFSSQLSNNMGMILFILVLYGLVGFLDDFLKVF
RKINEGLNPKQKLALQLLGGVIFYLFYERGGDMLSVFGYQVHLGIFYIVFALFWLVGF
SNAVNLTDGVDGLASISVVISLSAYGVIAYVQGQMDILLVILAMIGGLLGFFVFNHKP
AKVFMGDVGSLALGGMLAAISMALHQEWTLLIIGIVYVFETTSVMMQVSYFKLTGGKR
IFRMTPVHHHFELGGLSGKGNPWSEWKVDFFFWGVGLLASLLILAILYLM"
     gene                112328..114433
                         /gene="clpC_1"
                         /locus_tag="SAMEA3381655_00712"
     CDS                 112328..114433
                         /gene="clpC_1"
                         /locus_tag="SAMEA3381655_00712"
                         /inference="ab initio prediction:Prodigal:2.60"
```

```
                     /inference="similar to AA
                     /codon_start=1
                     /transl_table=11
                     /product="Group II intron maturase"
                     /protein_id="VRO31250.1"
/translation="MNNNFNNFNNMDDLFNQLMGGMRGYSSENRRYLINGREVTPEEF
AHYRATGQLPGNAETDVQMPQQASGMKQDGVLAKLGRNLTAEAREGKLDPVIGRNKEI
QETSEILSRRTKNNPVLVGDAGVGKTAVVEGLAQAIVNGDVPAAIKNKEIISIDISGL
EAGTQYRGSFEENVQNLVNEVKEAGNIILFFDEIHQILGAGSTGGDSGSKGLADILKS
ALSRGELTVIGATTQDEYRNTILKNAALARRFNEVKVNAPSAENTFKILQGIRDLYQQ
HHNVILPDEVLKAAVDYSVQYIPQRSLPDKAIDLVDVTAAHLAAQHPVTDVHAVEREI
ETEKDKQEKAVEAEDFEAALNYKTRIAELEKKIENHTEDMKVTASVNDVAESVERMTG
IPVSQMGASDIERLKDMAHRLQDKVIGQDKAVEAVARAIRRNRAGFDEGNRPIGSFLF
VGSTGVGKTELAKQLALDMFGTQDAIIRLDMSEYSDRTAVSKLIGTTAGYVGYDDNSN
TLTERVRRNPYSIILLDEIEKADPQVITLLLQVLDDGRLTDGQGNTVNFKNTVIIATS
NAGFGYEANLTEDADKPELMDRLKPFFRPEFLNRFNAVIEFSQLTKEDLSKIVDLMLA
EVNQTLAKKDIDLVVSQAAKDYITEEGYDEVMGVRPLRRVVEQEIRDKVTDFHLDHLD
AKHLEADMKDGGLVIREKS"
     gene            complement(114834..115316)
                     /gene="luxS"
                     /locus_tag="SAMEA3381655_00713"
     CDS             complement(114834..115316)
                     /gene="luxS"
                     /locus_tag="SAMEA3381655_00713"
                     /EC_number="4.4.1.21"
                     /inference="ab initio prediction:Prodigal:2.60"
                     /inference="similar to AA
sequence:RefSeq:YP_001449908.1"
                     /codon_start=1
                     /transl_table=11
                     /product="S-ribosylhomocysteinase"
                     /protein_id="VRO31253.1"
/translation="MSKEVIVESFELDHTIVKAPYVRLIGEETGPKGDIISNYDIRLV
QPNEDSIPTAGLHTIEHLLAKLIRTRIDGMIDCSPFGCRTGFHMIMWGRHTSAKIAAV
IKDSLKEIAETTTWEDVPGTTIESCGNYKDHSLFSAKEWAKLILEQGISDDAFERHVI
                     "
     gene            complement(115411..116895)
                     /locus_tag="SAMEA3381655_00714"
     CDS             complement(115411..116895)
                     /locus_tag="SAMEA3381655_00714"
                     /inference="ab initio prediction:Prodigal:2.60"
                     /inference="similar to AA
sequence:RefSeq:YP_003724085.1"
                     /codon_start=1
                     /transl_table=11
                     /product="ATP-dependent Zn protease"
                     /protein_id="VRO31256.1"
/translation="MKKQAFSSEQYLNLQRDHILERINQFDGKLYLEFGGKMLEDFHA
ARVLPGYEPDNKIKLLQELKEQVEVVIAINASNIEHSKARGDLGISYDQEVLRLIDKF
NELGIFVGSVVITQYAGQPAADAFRNQLEKNGIDSYLHYPIKGYPTDMDHIISPEGMG
KNDYIKTSRNLIVVTAPGPGSGKLATCMSNMYHDQINVIKSGYAKFETFPVWNLPLHH
PVNLAYEAATADLDDVNMIDPFHLQTYGETTVNYNRDIEIFPVLKRMLERILGKSPYA
SPTDMGVNMVGFAITDDEAAVEASKQEIIRRYYQTVLDFKAEKVGEAAVKKIELLMND
LGITPADRKVAVVARQKAEETGGPALALELPNGDIVTGKNSELFGPTAAALINAIKKS
```

```
ADIAKEVKLIEPEVVKPIQGLKIDHLGSRNPRLHSNEILIALAITATENPDAARAMEE
LGNLKGSEAHSTIILTDEDKNVLRKLGINVTFDPYYQYDRLYRK"
     gene            117048..118658
                     /gene="dexB"
                     /locus_tag="SAMEA3381655_00715"
     CDS             117048..118658
                     /gene="dexB"
                     /locus_tag="SAMEA3381655_00715"
                     /EC_number="3.2.1.70"
                     /inference="ab initio prediction:Prodigal:2.60"
                     /inference="similar to AA
sequence:RefSeq:YP_004326655.1"
                     /codon_start=1
                     /transl_table=11
                     /product="glucan 1,6-alpha-glucosidase"
                     /protein_id="VRO31259.1"
/translation="MQEKWWHNAVVYQVYPKSFMDSNGDGVGDLPGITSKLDYLAKLG
ITAIWLSPVYDSPMDDNGYDIVDYQAIATIFGTMEDMDQLIAEAKKRGIRIIMDLVVN
HTSDEHAWFVEACENTDSPERDYYIWRDEPNDLDSIFSGSAWEYDEKSGQYYLHFFSK
KQPDLNWENEKLRQKIYEMMNFWIDKGIGGFRMDVIDMIGKIPDEKVVNNGPMLHPYL
KEMNQATFGDKNLLTVGETWGATPEIAKLYSDPKGQELSMVFHFEHIGLQYQEGQPKW
HYQKELNIAKLKEIFNKWQTELGVEDGWNSLFWNNHDLPRIVSIWGNDQEYREKSAKA
FAILLHLMRGTPYIYQGEEIGMTNYPFETLDQVEDIESINYAREALEKGVPMQEIMDS
IRVIGRDNARTPMQWDENKNAGFSTGQPWLAVNPNYQAINVQEALANPDSIFYTYQKL
VQIRKENSWLIRSDFELLETADKVFAYIRKDGDRRFLVVANLSNEEQDLIVEGNVKSV
                     LIENTAAQEVFEKQILAPWDAFCVELTD"
     gene            118934..120901
                     /gene="aliB1"
                     /locus_tag="SAMEA3381655_00716"
     CDS             118934..120901
                     /gene="aliB1"
                     /locus_tag="SAMEA3381655_00716"
                     /inference="ab initio prediction:Prodigal:2.60"
                     /inference="similar to AA
sequence:RefSeq:YP_004326654.1"
                     /codon_start=1
                     /transl_table=11
                     /product="oligopeptide ABC transporter AliB-like
protein"
                     /protein_id="VRO31262.1"
/translation="MQSKKWLLGAGVTLTTALLLTACGKSEKNADAPKTFSYVYAVDP
SSLDYSVTSKSSTSDVIGNVVDGLLENDKYGDLIPSLAEDWSVSKDGLTYTYKLRKGV
KWYTSEGEEYAEVKAQDFVTGLKHAADGKSDGLSLVEKSIKGLEAYVSGETNDFSTVG
VKALDDYTVEYTLNQPESFWNSKVTTATMLPVNEEFLNATGKDYGAPTPSSILYNGPY
FLKSLISKSVIEYEKNPNYWDKENVKIDNVKLTFYDGSDQESLIRSFTQGAYTTARLF
PASSNFESTKKEYGDKIVYSPQEATSYYLTVNVNRQSYNKTAKTDESQKTSTKEALLN
KNFRQALNFAFNRHAYTAQLNGEEGADKIIRNSLVPDNYVQVAGKTFGQLAQDELLKY
GEQWKDVTLTDGKDTIYNPTKAKSAFEKAKSELQAKGVSFPIHLDVPVEQTDVVAVQQ
TNSLKQSIEETLGTENVIIDVLQMTDNEKESITSQAKVPTQKDYDLNGTGWGPDYQDP
ATYLNILDAKKGSALKHLGITKGKDPEVVAKVGLDEYKNLLDDAASETSNLDKRYEKY
AKAQAWVTDSSLLIPVASSGGSPMVSRTVPFTKAYSQVGIKGDPFIFKGMELQNDIVT
TKEYEAAFKKWQKEKLESNAQYQKDLEKHVK"
     gene            121105..122811
                     /gene="aliB2"
```

```
                     /locus_tag="SAMEA3381655_00717"
     CDS             121105..122811
                     /gene="aliB2"
                     /locus_tag="SAMEA3381655_00717"
                     /inference="ab initio prediction:Prodigal:2.60"
                     /inference="similar to AA
sequence:RefSeq:YP_004326653.1"
                     /codon_start=1
                     /transl_table=11
                     /product="AliB-like protein"
                     /protein_id="VRO31265.1"
/translation="MNTKRRVLSTGLTFVAALLLAACGQSGSDTKTYSSTFSGNPTTF
NYLLDYYADNTSIITNLVDGLLENDNYGNLVPSLAEDWSVSSDGLTYTYKLRKDAKWF
TADGEEYAPVKAQDFVTGIKYAVDNKSQAIDLIQNSIKGLNDYIIGADSDFSKVGVKA
IDDQTVEYTLTRPEPYWNSKTTNSILFPVNEEFLNSKGKDFGTLSPDSILYSGPYLLK
DFTSKSSIEYVKNPHYYDHDKVSIEHVKLAYFDGSDQELTIRNFESGAYSIAGVYPNS
SNFAKTKEKYKDNIVYSLQDKTSWYFNFNVNRKAYNHTAKTTDEQKKSTETAVLNKNF
RQAVNFALDRTAYSAQSNGEEAASKTLRNTLVPSTFVQVGDKTFGEVVASKLVNYGTE
WSGINLEDTQDAYFNKEKAQAKFAEAKKELTSQGVTFPIHLDVAVDQTSKNAVTGMNS
VKQTLESVLGADNIVIDVQQLSTDDFNNVAFLAPTAADRDYDLNFDGWVGDYQDPSTY
LNPFNAEDGFYLKIFGLDAKEDKEKITSLGLDTYTKMLKDAHSENKDVAKRYEKYAEA
QA"
     gene            122812..123063
                     /gene="sarA_1"
                     /locus_tag="SAMEA3381655_00718"
     CDS             122812..123063
                     /gene="sarA_1"
                     /locus_tag="SAMEA3381655_00718"
                     /inference="ab initio prediction:Prodigal:2.60"
                     /inference="similar to AA
sequence:RefSeq:YP_004767777.1"
                     /codon_start=1
                     /transl_table=11
                     /product="AliB-like protein"
                     /protein_id="VRO31268.1"
/translation="MIDNSLVMSAMSSGGTASVTKVTPFTRGYSLVGIKGDGDNYKYM
KLQKDTVTTKQFEEAKTKWEQESKKAIEKAQKEAEKHVK"
     gene            123386..123787
                     /gene="glf_1"
                     /locus_tag="SAMEA3381655_00719"
     CDS             123386..123787
                     /gene="glf_1"
                     /locus_tag="SAMEA3381655_00719"
                     /EC_number="5.4.99.9"
                     /inference="ab initio prediction:Prodigal:2.60"
                     /inference="similar to AA
sequence:RefSeq:YP_004326638.1"
                     /codon_start=1
                     /transl_table=11
                     /product="UDP-galactopyranose mutase"
                     /protein_id="VRO31271.1"
/translation="MYDYLIVGAGLSGAIFAYEATKRGKKVKVIDKRDHIGGNIYCEN
VEGINVHKYGAHIFHTSNKKVWNYVNQFAEFNNYINSPLANYKGSLYNLPFNMNTFYA
MWGTKTPQEVKDKIAEQTAHMKDVEPKNLEE"
```

```
     gene            123797..124303
                     /gene="glf_2"
                     /locus_tag="SAMEA3381655_00720"
     CDS             123797..124303
                     /gene="glf_2"
                     /locus_tag="SAMEA3381655_00720"
                     /EC_number="5.4.99.9"
                     /inference="ab initio prediction:Prodigal:2.60"
                     /inference="similar to AA
sequence:RefSeq:YP_004326638.1"
                     /codon_start=1
                     /transl_table=11
                     /product="UDP-galactopyranose mutase"
                     /protein_id="VRO31274.1"
/translation="MIGSDIYEKLIKGYTEKQWGRSATDLPPFIIKRLPVRLTFDNNY
FNDRYQGIPIGGYNVIIENMMKDVEVELGLNFFANRQELEASAEKVVFTGMIDQYFDY
KHGELEYRSLRFEHKVLHEENYQGNAVVNYTEREIPYTLRKSLQTASTLPYLQPQSSA
LSNLQLAS"
     gene            complement(124415..124786)
                     /gene="doc"
                     /locus_tag="SAMEA3381655_00721"
     CDS             complement(124415..124786)
                     /gene="doc"
                     /locus_tag="SAMEA3381655_00721"
                     /inference="ab initio prediction:Prodigal:2.60"
                     /inference="similar to AA
sequence:RefSeq:YP_008501405.1"
                     /codon_start=1
                     /transl_table=11
                     /product="Death on curing protein"
                     /protein_id="VRO31277.1"
/translation="MKRLTTEQVLALHRQLIVASGGMDGIRDKGLVESSLSNVFDTYF
EVEQYLTIEEKAARLCYSLIKNHAFLDGNKRIGIFVMLVLLEINDIVLDCTDEELVHL
GLGVATSKLTYEDILDFVKNH"
     gene            complement(124783..125040)
                     /locus_tag="SAMEA3381655_00722"
     CDS             complement(124783..125040)
                     /locus_tag="SAMEA3381655_00722"
                     /inference="ab initio prediction:Prodigal:2.60"
                     /inference="similar to AA
sequence:RefSeq:YP_059809.1"
                     /codon_start=1
                     /transl_table=11
                     /product="cytoplasmic protein"
                     /protein_id="VRO31280.1"
/translation="MQINLENLVSITEANQNFSKVARMVDSKGTAVILKNNKPKYILV
DYNTLIQEEQTEAIIADQTGVDEVANSILSRHLEAFKELAK"
     gene            125259..127238
                     /gene="aliA"
                     /locus_tag="SAMEA3381655_00723"
     CDS             125259..127238
                     /gene="aliA"
                     /locus_tag="SAMEA3381655_00723"
                     /inference="ab initio prediction:Prodigal:2.60"
```

```
                     /inference="similar to AA
sequence:RefSeq:YP_001835080.1"
                     /codon_start=1
                     /transl_table=11
                     /product="oligopeptide ABC transporter
                     oligopeptide-binding protein AliA"
                     /protein_id="VRO31283.1"
/translation="MKTRKVLALVGVTLLAAGVLAACSGGSGAQGEKTFAFTYETHPD
NLNYLTTGKAATSEITSNVIDGLLENDKYGNLVPSLAEDWSVSKDGLTYTYKIRQDAK
WYTSEGEEYALVKAQDFVTGLKYATDKKADALYLVQDSIKGLDAYAKGEITDFAQVGI
KALDDQTVQYTLNKPETFWNSKTTMGVLAPVNEEFLNSKGDDFAKATDPSSLLYNGPY
LLKSIVTKSSVEFAKNPNYWDKDNVHIDKVKLSFWDGQDTSKPAENFKDGSLTAARLY
PTSASFAELEKSMKDNIVYTQQDSITYLVGTNIDRQSYKYTSKTSEEQKTSTKKALLN
KDFRQAIAFGFDRTAYASQLNGQTGASKILRNIFVPPTFVQADGKNFGELVKEKLVTY
GDEWKDVNLADSQDGLYNPEKAKAEFAKAKSALQAEGVTFPIHLDMPVDQTATTKVQR
VQSMKQSLEATLGADNVIIDIQQLQKDEVNNITYFAENAAGEDWDLSDNVGWGPDFAD
PSTYLDIIKPSVGESTKTYLGFDSGEDNVAAKKVGLYDYEKLVTEAGDEATDVAKRYD
KYAAAQAWLTDSALIIPTTSRTGRPILSKIVPFTIPFALSGNKGTSDPVLYKYLEIQD
KAVTADEYQKAQEKWMKEKAESNKKAQEELAKHVK"
     gene            complement(127612..129768)
                     /gene="pbp1A"
                     /locus_tag="SAMEA3381655_00724"
     CDS             complement(127612..129768)
                     /gene="pbp1A"
                     /locus_tag="SAMEA3381655_00724"
                     /inference="ab initio prediction:Prodigal:2.60"
                     /inference="similar to AA
sequence:RefSeq:YP_006702064.1"
                     /codon_start=1
                     /transl_table=11
                     /product="penicillin-binding protein 1A"
                     /protein_id="VRO31286.1"
/translation="MKKPTILRLLKYLSISFLSLVIAAIVLGGGVFFYYVSKAPSLSE
SKLVATTSSKIYDNKNQLIADLGSERRVNAQANDIPTNLVKAIVSIEDHRFFDHRGID
TIRILGAFLRNLQSNSLQGGSTLTQQLIKLTYFSTSTSDQTISRKAQEAWLAIQLEQK
ATKQEILTYYINKVYMSNGNYGMQTAAQNYYGKDLNNLSLPQLALLAGMPQAPNQYDP
YSHPKAAQDRRNLVLSEMKNQGYISAEQYEKAVNTPITDGLQSLKSASNYPAYMDNYL
KEVINQVEEETGYNLLTTGMDVYTNVDQEAQKHLWDIYNTDEYVAYPDDELQVASTIV
DVSNGKVIAQLGARHQSSNVSFGINQAVETNRDWGSTMKPITDYAPALEYGVYDSTAT
IVHDEPYNYPGKDIPVYNWDRGYFGNITLQYALQQSRNVPAVETLNKVGLNRAKTFLN
GLGIDYPSIHYSNAISSNTTESNKKYGASSEKMAAAYAAFANGGTYYKPMYIHKVVFS
DGSEKEFSNVGTRAMKETTAYMMTDMMKTVLTYGTGRNAYLAWLPQAGKTGTSNYTDE
EIENHIKTSQFVAPDELFAGYTRKYSMAVWTGYSNRLTPLVGNGLTVAAKVYRSMMTY
LSEGSNPEDWNIPEGIYRNGQFVFQNGARPTWTETTSQPSSTESSSTSTESSTSQAPT
TSPDASTNGPNQAPNSPGANQNPAPQTPQVQPRPQQ"
```

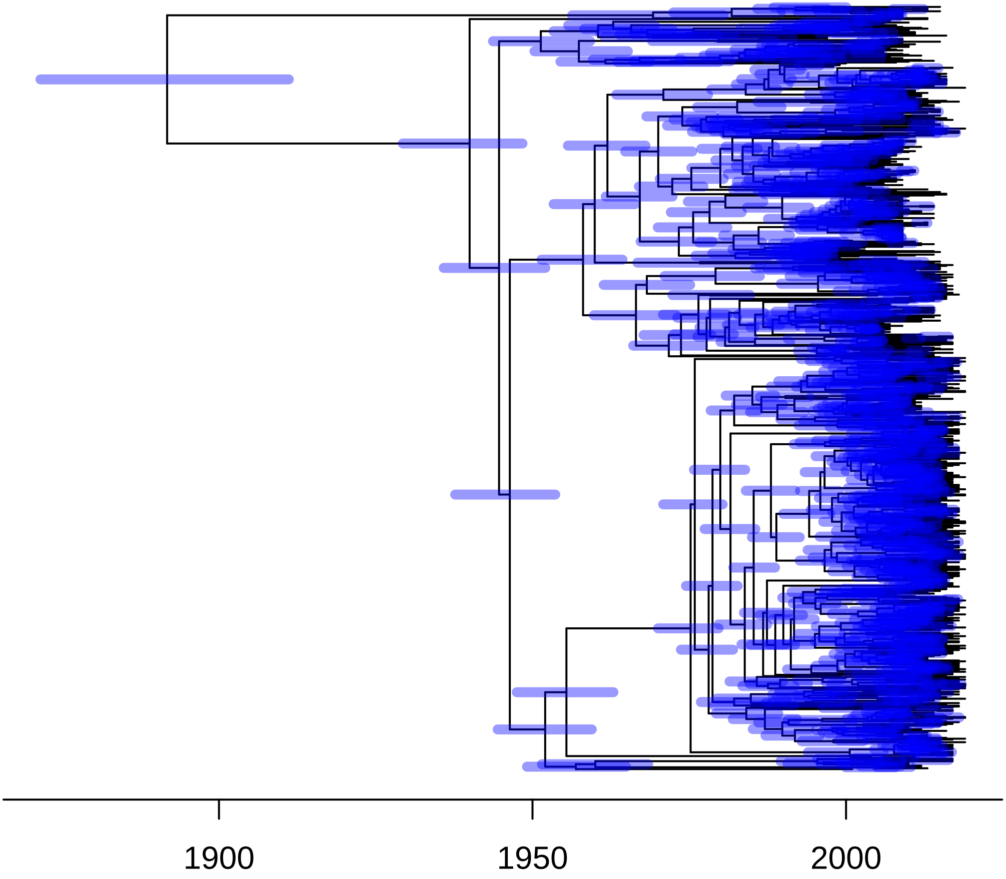
1900
1950
2000
